# REMOVER-PITCh: microhomology-assisted long-range gene replacement with highly multiplexed CRISPR-Cas9

**DOI:** 10.1007/s11626-024-00850-1

**Published:** 2024-02-09

**Authors:** Shu Matsuzaki, Tetsushi Sakuma, Takashi Yamamoto

**Affiliations:** 1 Division of Integrated Sciences for Life, Graduate School of Integrated Sciences for Life, Hiroshima University, 1-3-1 Kagamiyama, Higashi-Hiroshima, Hiroshima 739-8526 Japan; 2 Drug Discovery Laboratory, Wakunaga Pharmaceutical Co., Ltd., 1624 Shimokotachi, Koda-Cho, Akitakata-Shi, Hiroshima 739-1195 Japan

**Keywords:** CRISPR-Cas9, Genome editing, Gene replacement, Microhomology-mediated end joining

## Abstract

A variety of CRISPR-Cas9-based gene editing technologies have been developed, including gene insertion and gene replacement, and applied to the study and treatment of diseases. While numerous studies have been conducted to improve the efficiency of gene insertion and to expand the system in various ways, there have been relatively few reports on gene replacement technology; therefore, further improvements are still needed in this context. Here, we developed the REMOVER-PITCh system to establish an efficient long-range gene replacement method and demonstrated its utility at two genomic loci in human cultured cells. REMOVER-PITCh depends on microhomology-assisted gene insertion technology called PITCh with highly multiplexed CRISPR-Cas9. First, we achieved gene replacement of about 20-kb *GUSB* locus using this system. Second, by applying the previously established knock-in-enhancing platform, the LoAD system, along with REMOVER-PITCh, we achieved the replacement of a longer gene region of about 200 kb at the *ARSB* locus. Our REMOVER-PITCh system will make it possible to remove and incorporate a variety of sequences from and into the genome, respectively, which will facilitate the generation of various disease and humanized models.

**Supplementary Information:**

The online version contains supplementary material available at 10.1007/s11626-024-00850-1.

## Introduction

To date, genome editing technology has led to the rapid development of genetically engineered cells in various fields such as medicine, basic biology, and bioengineering. Genome editing is a technology that can modify the target genome typically by introducing DNA double-strand breaks (DSBs) in a site-specific manner by utilizing programmable nucleases and endogenous DSB repair mechanisms (Sakuma and Woltjen 2014). If an exogenous DNA molecule with the optimized structure described below is provided along with genome editing tools, a foreign gene sequence can be inserted into the target site (i.e., gene knock-in) (Sakuma and Yamamoto 2017).

Of many DSB repair mechanisms, the following three pathways have been widely used in the context of plasmid DNA-based gene knock-in: non-homologous end joining (NHEJ) (Maresca *et al*. 2013; Suzuki *et al*. 2016), homologous recombination (HR) (Hockemeyer *et al*. 2009; Baker *et al*. 2017), and microhomology-mediated end joining (MMEJ) (Nakade *et al*. 2014; Sakuma *et al*. 2015; Sakuma *et al*. 2016b). For the NHEJ-mediated knock-in, a cassette containing the sequence of interest is directly located next to the cleavage sequence on the donor vector without homology arms (Suzuki *et al*. 2016). For gene knock-in via the HR pathway, around 1-kb sequences upstream and downstream of the genomic cleavage site should be added to the donor vector as the homology arms, which enable accurate knock-in (Hockemeyer *et al*. 2009). The MMEJ repair pathway-mediated gene knock-in approach has been reported as PITCh (Precise Integration into Target Chromosome) system (Nakade *et al*. 2014; Sakuma *et al*. 2015; Sakuma *et al*. 2016b; Nakamae *et al*. 2017), in which an exogenous cassette linearized in living cells can be knocked into the target site via a short microhomologies up to around 40 bp. Since MMEJ repair is highly active in a majority of cell cycles in various species, the PITCh system can efficiently be applied not only in cultured cells but also in various animals including mice and zebrafish (Hisano *et al*. 2015; Aida *et al*. 2016; Nakagawa *et al*. 2017).

The CRISPR-Cas9 system is an RNA-guided nuclease derived from the acquired immune system of bacteria (Wiedenheft *et al*. 2012). CRISPR-Cas9 requires a single-guide RNA (sgRNA) containing a complementary sequence to the target DNA of about 20 bases and a Cas9 protein with nuclease activity, where a protospacer-adjacent motif (PAM) sequence serves as an indicator of cleavage by Cas9 (Cong *et al*. 2013; Mali *et al*. 2013). In this study, we use SpCas9 from *Streptococcus pyogenes*, which uses 5′-NGG-3′ as the PAM sequence and cleaves three bases upstream of the PAM (Cong *et al*. 2013; Mali *et al*. 2013). CRISPR-Cas9 has been widely used as a very simple and flexible genome editing tool because it can target any genomic region of various cells and organisms by simply producing custom sgRNAs. It is also capable of simultaneous disruption of many genes or deletion of a genomic region by introducing or expressing multiple sgRNAs simultaneously (Wang *et al*. 2013; Sakuma *et al*. 2014; Sakuma *et al*. 2016a).

Gene replacement is a technology that removes a targeted gene region and replaces it with an intended sequence such as an exogenous gene cassette, typically using double-cut CRISPR-Cas9 strategy with dual-sgRNAs (Zheng *et al*. 2014; Danner *et al*. 2021). Gene replacement via HR repair using CRISPR-Cas9 has been frequently reported in mice and mouse embryonic stem (mES) cells, including examples of the removal of cluster genes and cassette replacement of gene regions of 25 to 65 kb (Zhang *et al*. 2015; Leidy-Davis *et al*. 2018). In addition, a recent study of the UKiS method showed up to 290-kb deletion with a single-cut CRISPR-Cas9 strategy in human cells (Ohno *et al*. 2022). However, although gene knock-in via HR repair is highly accurate, the HR-mediated strategy possesses limited applicability due to the narrow range of the HR activity during the late S/G2 phases in the cell cycle and the fact that its activity varies by organism and cell type (Mao *et al*. 2008). Gene replacement using MMEJ repair with dual-sgRNAs has also been reported, but it only demonstrated the replacement of a partial region on the gene (Aida *et al*. 2016; Nakagawa *et al*. 2017; Katayama *et al*. 2019). Therefore, the development of a replacement technology that is more efficient and can be applied to gene regions of longer lengths will increase the flexibility in the creation of humanized mice and disease models, and disease treatment.

In this study, we develop a novel gene replacement method, named REplacement with Multiplex OVERdigestion (REMOVER)-PITCh system, to extend the applicability and improve the efficiency of gene replacement technology. We selected *GUSB* and *ARSB* as the target loci, which encode the enzymes responsible for mucopolysaccharidosis types VII and VI, respectively (Linker *et al*. 1955; Valayannopoulos *et al*. 2010). These diseases are caused by the accumulation of the substrate glycosaminoglycans (GAGs) in the body as a result of the inability to supply enzymes with normal activity due to mutations introduced on both alleles of the corresponding genes. To date, tens to hundreds of pathogenic mutations have been identified in these diseases, depending on the disease type (Tomatsu *et al*. 2009; Tomanin *et al*. 2018). The gene replacement method can be a powerful approach to the allelic repair of diseases in which such a large number of pathogenic mutations are scattered on the corresponding genes, and also to the creation of animal models; therefore, we aimed to establish the REMOVER-PITCh system.

## Materials and methods

### Cell culture

HCT116 cells were maintained in Dulbecco’s modified Eagle’s medium (DMEM)—high glucose supplemented with 10% fetal bovine serum (FBS, Thermo Fisher Scientific, Waltham, MA), 1% penicillin–streptomycin (Wako, Richmond, VA), and 1% MEM non-essential amino acids (Thermo Fisher Scientific). Cells were tested negative for mycoplasma contamination using e-Myco Mycoplasma PCR Detection Kit (iNtRON Biotechnology, Seongnam, Korea) and were authenticated by short tandem repeat analysis (Takara, Shiga, Japan).

### Construction of multiplex CRISPR vectors

The multiplex CRISPR vectors for each locus were constructed using the Multiplex CRISPR/Cas9 Assembly System Kit (no. 1000000055, Addgene) (Sakuma *et al*. 2014). Briefly, oligonucleotides for sgRNA were synthesized and annealed, and the annealed oligos were inserted into the pX330A or pX330S vectors. To construct multiplex CRISPR vectors for the LoAD system, modified pX330A and pX330S vectors harboring the sgRNA expression cassettes containing the MS2 stem-loops were used. A list of the oligonucleotides used to generate the sgRNA cassettes is shown in Supplementary Table 1.

### Construction of PITCh donor vectors

PITCh donor vectors containing the cassettes replacing the target regions of the *ARSB* and *GUSB* loci were constructed using PCR and In-Fusion cloning (Takara). The sequences of the donor vectors are shown in Supplementary Sequence.

### Transfection for REMOVER-PITCh

1 × 10^5^ HCT116 cells were seeded into 24-well plates. After 24 h, a total of 450 ng of plasmids (150 ng of Multiplex CRISPR vector no. 1, 150 ng of Multiplex CRISPR vector no. 2, and 150 ng of PITCh donor vector for the *ARSB* or *GUSB* locus) were introduced into the cells using Lipofectamine LTX (Thermo Fisher Scientific).

### Transfection for REMOVER-PITCh using LoAD system

The day before transfection, 1 × 10^5^ HCT116 cells were seeded into 24-well plates. The next day, a total of 400 ng of plasmids (100 ng of Multiplex CRISPR vector no. 1, 100 ng of Multiplex CRISPR vector no. 2, and 100 ng of PITCh donor vector for the *ARSB* or *GUSB* locus, and 100 ng of MS2-CtIP vector) were introduced into the cells using Lipofectamine LTX (Thermo Fisher Scientific) and transferred to 6-well plates the next day.

### Neomycin selection and single-cell cloning

Three days after transfection, the culture medium was replaced with a medium containing G418 (800 μg/mL) to start drug selection. The medium was changed daily with G418-containing medium. After approximately 10 d of G418 selection, single-cell cloning was performed using a limiting dilution method. Briefly, diluted cells (6.0 cells/mL) were prepared from the post-selection cell population and 200 µL of diluted cells was added to each well of a 96-well plate (1.2 cells/well).

### Genomic PCR

Genomic DNA was extracted from knock-in cell populations or knock-in clones using DNeasy Blood & Tissue Kit (Qiagen, Hilden, Germany). Genomic PCR was performed using KOD FX Neo (Toyobo, Osaka, Japan) with primers listed in Supplementary Table 2. PCR products were separated by agarose gel electrophoresis and the amplified bands were observed using a UV transilluminator after EtBr staining.

### Sequencing analysis

Sequencing analysis was performed using SeqStudio Genetic Analyzer (Thermo Fisher Scientific) with BigDye Terminator v3.1 Cycle Sequencing Kit (Life Technologies, Carlsbad, CA). The PCR reaction was performed using a thermal cycler at 96°C for 2 min → (96°C for 10 s → 50°C for 5 s → 60°C for 4 min) × 25 → 4°C for ∞. PCR products were purified by ethanol precipitation, dissolved in Hi-DI Formamide (Thermo Fisher Scientific), heat-treated at 95°C for 2 min, cooled on ice for 5 min, and then subjected to cycle sequencing.

### Off-target analysis

Seven potential off-target candidate sites were selected using the COSMID software (https://crispr.bme.gatech.edu/). Genomic DNA for PCR amplification of the off-target candidate sites was extracted from knock-in clones using DNeasy Blood & Tissue Kit (Qiagen). PCR amplification of each region was performed using KOD One (Toyobo) or PrimeStar GXL (Takara) with primers listed in Supplementary Table 3. Mutation analysis at each off-target candidate site was performed by sequencing analysis and Cel-I assay using GeneArt Genomic Cleavage Detection Kit (Life Technologies).

## Results and discussion

### Design of REMOVER-PITCh

To start with, we performed the trial of standard PITCh-mediated gene replacement at the *GUSB* and *ARSB* loci in HCT116 cells, in which CRISPR-Cas9 with dual-sgRNAs was used along with the PITCh donor vector (Supplementary Fig. 1*a*-*c*, *g*). At the *GUSB* locus, genotyping analysis in the isolated clones showed that none of them had the intended alleles replaced with the *GUSB* gene cassette (Supplementary Fig. 1*d*, *e*). In addition, sequencing analysis revealed that a frameshift mutation was caused by a single base insertion (Supplementary Fig. 1*f*). Similarly, at the *ARSB* locus, no clones showed the objective amplicons by out-out PCR analysis, although the knock-in junctions were correctly jointed via MMEJ (Supplementary Fig. 1*h*-*j*). These results suggested that unpredicted and unintended imperfect replacement occurred. To clarify this, we performed PCR amplification from 5′ or 3′ UTR to each exon to investigate which region of the cassette was inserted into the genome. As a result, the amplification in the region from the 5′ UTR to exon 3 and 4 was confirmed, but that from the 5′ UTR to exon 5 was not observed on the 5′ side (Supplementary Fig. 1*k*). On the 3′ side, it was suggested that the insertion of a partial cassette fragment (*ARSB* CDS exon 8-T2A-NeoR) occurred (Supplementary Fig. 1*l*). These observations suggest that unintended homology-directed repair (HDR) may occur between the donor cassette and the genomic DNA via exon 4 and exon 8 sequences, resulting in imperfect knock-in (Supplementary Fig. 1*m*). In fact, a similar phenomenon was observed in previous studies showing combinatorial knock-in via NHEJ and HDR (Suzuki *et al*. 2019; Yoshimi *et al*. 2021).

**Figure 1.**
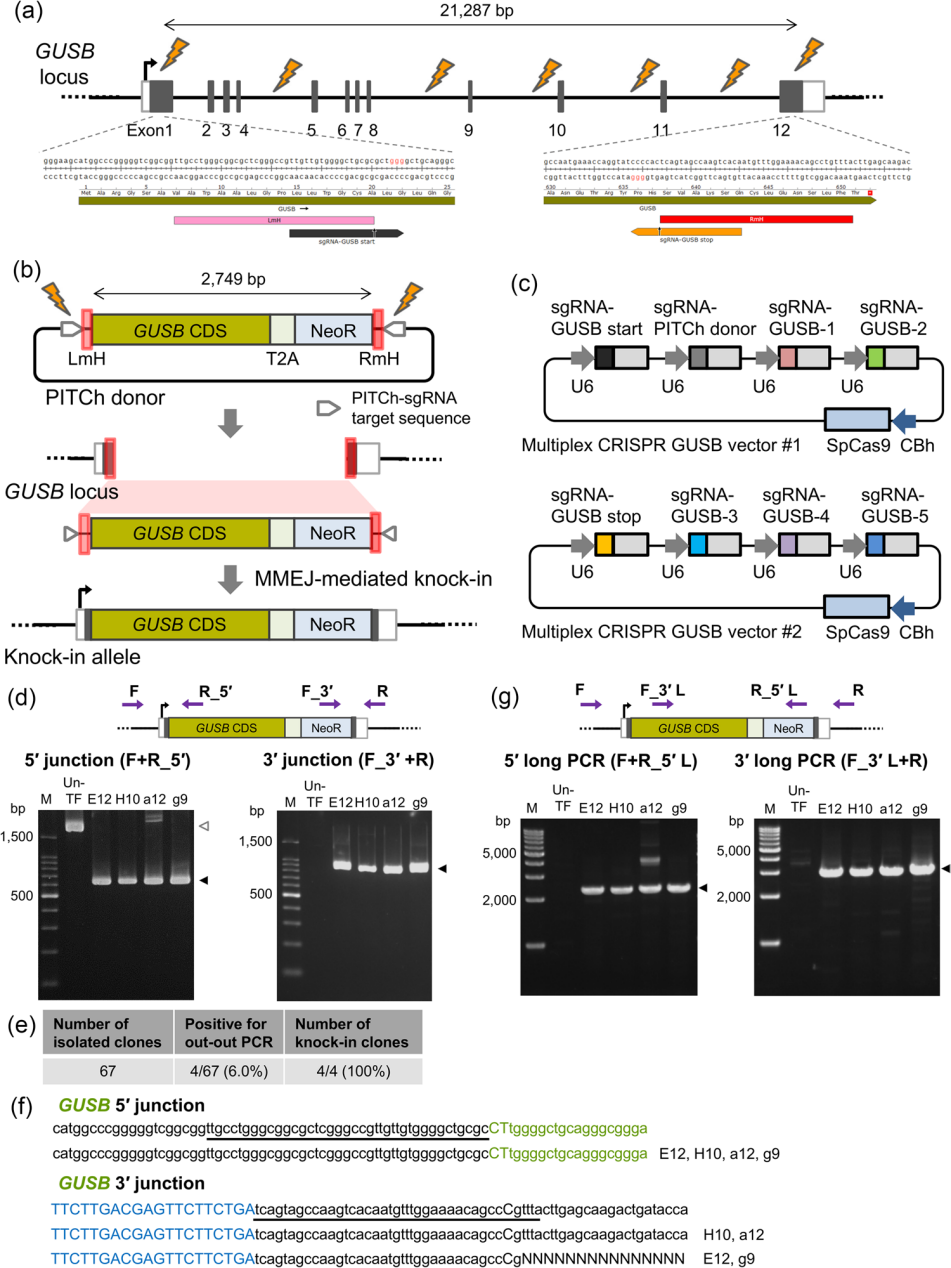
Validation of targeted replacement by REMOVER-PITCh at the *GUSB* locus. (*a*)Schematic illustration of the genomic context and the sgRNA target sites at the *GUSB* locus. *White* and *gray boxes* indicate 5′ and 3′ UTR, and exon, respectively. (*b*) Schematic illustration of the PITCh donor and the knock-in allele. The PITCh donor contains promoterless *GUSB* CDS-T2A-NeoR cassette flanked by 40-bp microhomologous sequences, which are indicated in *red boxes*. CDS, coding sequence. NeoR, neomycin resistance gene. LmH, left microhomologous sequence. RmH, right microhomologous sequence. (*c*) Schematic illustration of multiplex CRISPR vectors for the *GUSB* locus. *Colored boxes* excluding SpCas9 indicate the target sequence of each sgRNA. *Light gray boxes* indicate consensus sequences of sgRNA. U6, human U6 promoter. CBh, chicken beta-actin hybrid promoter. SpCas9, *Streptococcus pyogenes* Cas9. *d*, *g* Confirmation of targeted replacement in the isolated clones by genomic PCR. *Purple arrows* on the schematic illustration of the knock-in allele indicate primers for PCR amplification. Genotyping was performed by junction PCR (F + R_5′ and F_3′ + R) (*d*) and long PCR (F + R_5′L and F_3′L + R) (*g*). The clone IDs are indicated at the top of each gel image. *Black *and* white triangles* indicate the amplicon sizes of knock-in alleles and non-knock-in alleles, respectively. M, ladder marker. Un-TF, untransfected cells. (*e*) Table summarizing the number of knock-in clones established. (f) Sequencing analysis of knock-in junctions in the isolated clones by Sanger sequencing. The intended knock-in sequence is shown at the top of each sequence. *Green* and *blue letters* indicate the coding sequences of *GUSB* and NeoR, respectively. *Underlines* indicate microhomologous sequences. *N* indicates the undetermined bases due to the duplication of multiple waveforms.

**Figure 2.**
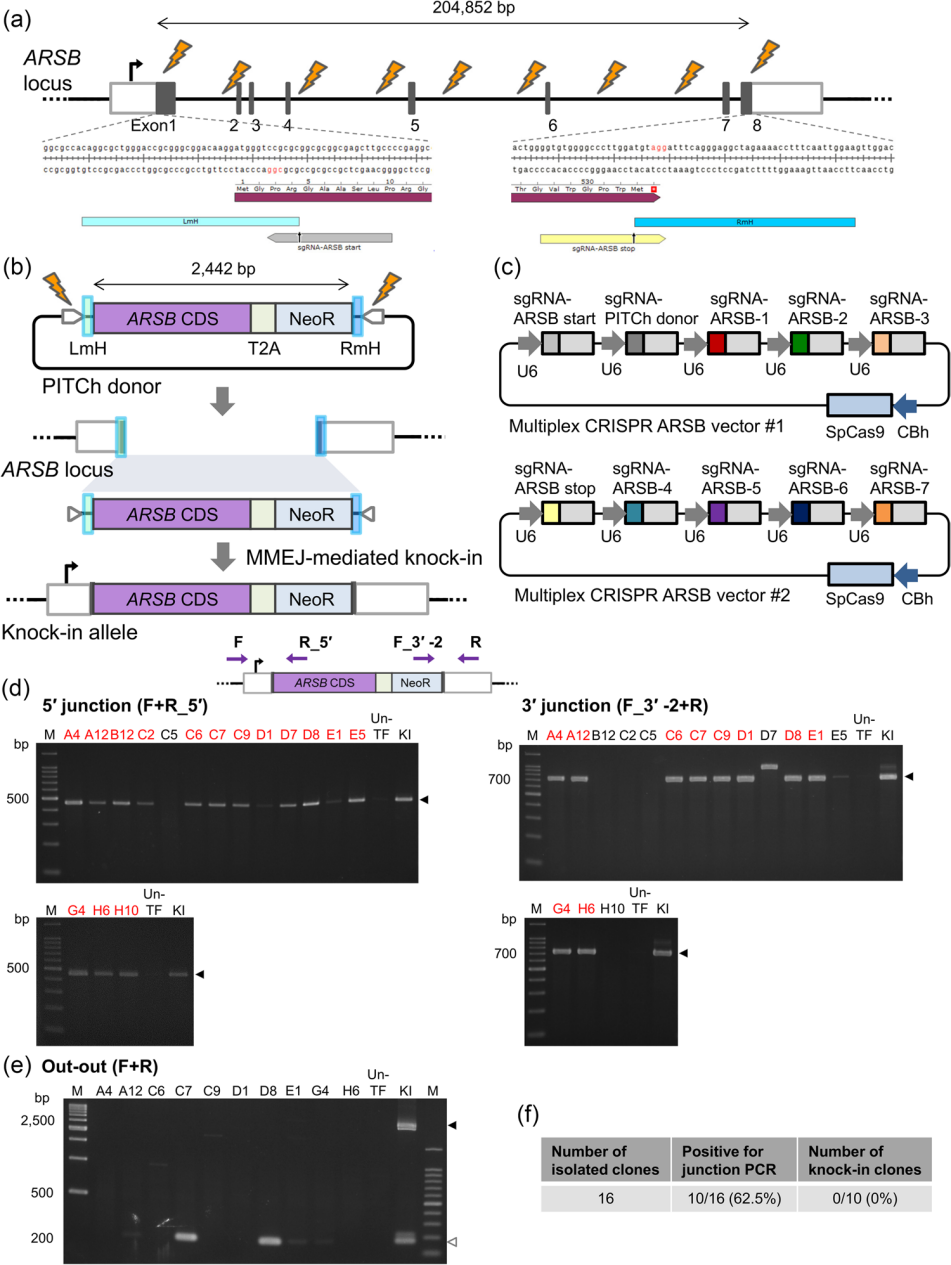
Validation of targeted replacement by REMOVER-PITCh at the *ARSB* locus. (*a*) Schematic illustration of the genomic context and the sgRNA target sites at the *ARSB* locus. *White* and *gray boxes* indicate 5′ and 3′ UTR, and exon, respectively. (*b*) Schematic illustration of the PITCh donor and the knock-in allele. The PITCh donor contains promoterless *ARSB* CDS-T2A-NeoR cassette flanked by 40-bp microhomologous sequences, which are indicated in *blue boxes*. CDS, coding sequence. NeoR, neomycin resistance gene. LmH, left microhomologous sequence. RmH, right microhomologous sequence. (*c*) Schematic illustration of multiplex CRISPR vectors for the *ARSB* locus. *Colored boxes* excluding SpCas9 indicate the target sequence of each sgRNA. *Light gray boxes* indicate consensus sequences of sgRNA. U6, human U6 promoter. CBh, chicken beta-actin hybrid promoter. SpCas9, *Streptococcus pyogenes* Cas9. (*d*, *e*) Confirmation of targeted replacement in the isolated clones by genomic PCR. *Purple arrows* on the schematic illustration of the knock-in allele indicate primers for PCR amplification. Genotyping was performed by junction PCR (F + R_5′ and F_3′-2 + R) (*d*) and out-out PCR (F + R) (*e*). The clone IDs are indicated at the top of each gel image. The clones that showed amplification of both junctions are indicated in *red letters*. *Black* and *white triangles* indicate the amplicon sizes of the knock-in and deleted alleles, respectively. M, ladder marker. Un-TF, untransfected cells. KI, Drug-selected cell populations. (*f* )Table summarizing the number of knock-in clones established.

Based on these observations, it was thought that the intervening genomic DNA possessing sequence homology with the donor cassette exerted a negative influence on gene replacement. Therefore, we attempted to develop an improved gene replacement method named REMOVER-PITCh, making the desired replacement more efficient. REMOVER-PITCh shreds and replaces a large genomic region with the donor cassette via MMEJ repair, by introducing multiple cleavages specifically in the intervening genomic region by targeting intronic sequences (Figs. 1a, *b*, and 2*a*, *b*). In this study, we confirmed the utility of REMOVER-PITCh at the *GUSB* and *ARSB* loci.

### REMOVER-PITCh at the GUSB locus in HCT116 cells

First, we designed a PITCh donor vector and multiplex CRISPR vectors targeting the *GUSB* locus (Fig. 1*b*, *c*). The *GUSB* gene encodes β-d-glucuronidase, the enzyme responsible for a congenital genetic disorder, mucopolysaccharidosis type VII, comprising a 21,287-bp gene in the genome. As the multiplex CRISPR vectors for the *GUSB* locus, we constructed two kinds of vectors each expressing four sgRNAs and SpCas9 (no. 1, no. 2) (Fig. 1*c*). Of these sgRNAs, five were designed to cleave the genomic intron for shredding, two were designed nearby the start and stop codons of the target gene to insert the donor cassette via MMEJ, and one was designed to cleave the PITCh donor. The PITCh donor vector consists of a human *GUSB* coding sequence fused with a T2A sequence, followed by a neomycin resistance gene (*GUSB* CDS-2A-NeoR). This cassette was flanked by 40-bp microhomologies that match the cleavage end sequences of the target gene. The *GUSB* CDS-2A-NeoR cassette does not contain promoter sequence; thus, the expression of the knocked-in cassette is induced by the endogenous *GUSB* promoter only when the cassette is incorporated into the target locus (Fig. 1*b*). Besides, silent mutations were introduced into the sgRNA target sequences on the coding sequence of *GUSB* in the PITCh donor to prevent the unintended donor cleavage (Supplementary Fig. 2*a*).

Next, we demonstrated REMOVER-PITCh at the *GUSB* locus in HCT116 cells. The constructed multiplex CRISPR and PITCh donor vectors were co-transfected into HCT116 cells, and 3 d after transfection, drug selection with G418 was performed. After selection and initial validation by genomic PCR analysis with the cell population, 67 cell clones were isolated by single cell cloning. Out of 67 clones, four clones (6.0%) showed the possibility of cassette replacement on at least one allele by out-out PCR (Supplementary Fig. 2*b*). Furthermore, genomic PCR analysis of 5′ and 3′ knock-in junctions showed the objective amplicons from these four clones (Fig. 1*d*, *e*). Sequencing analysis of the 5′ and 3′ knock-in junctions also showed an accurate knock-in sequence, indicating that the targeted genomic region was correctly replaced with the donor cassette via MMEJ (Fig. 1*f*, Supplementary Fig. 2*c*), although the possibility of mutations downstream of the right microhomology existed in E12 and g9 clones because overlapping peaks were observed in their sequencing data. In addition, we also performed 5′ and 3′ long PCR analyses to confirm the perfect knock-in, and found that the four clones showed knock-in allele-specific amplification (Fig. 1*g*). Based on these results, we demonstrated the utility of REMOVER-PITCh in human cultured cells and its applicability to replace a gene region of about 20 kb long.

To investigate whether the knock-in allele in the four clones was homozygous or heterozygous, non-knock-in allele-specific PCR amplification was performed. Of the four knock-in clones, clone a12 showed amplification of the region from 5′ UTR to intron 1, and clone H10 showed amplification of the region from intron 11 to 3′ UTR, suggesting the presence of the allele other than the perfect knock-in allele (Supplementary Fig. 3*a*, *b*). Sequencing analysis showed that there was no mutation in the 5′ cleavage site of clone a12. On the other hand, in clone H10, a single base-pair insertion at the 3′ cleavage site was observed (Supplementary Fig. 3*c*). From these results, we assumed that clones E12 and g9 were homozygous knock-in allele, while clones H10 and a12 were heterozygous knock-in allele, although we could not completely eliminate the possibility of the existence of the undetectable allele other than the perfect knock-in allele in E12 and g9 clones.

### REMOVER-PITCh at the ARSB locus in HCT116 cells

To investigate whether REMOVER-PITCh can be applied to larger gene replacement, we subsequently targeted the *ARSB* locus in HCT116 cells (Fig. 2*a*, *b*). The *ARSB* gene encodes arylsulfatase b, the enzyme responsible for mucopolysaccharidosis type VI, comprising a 204,852-bp gene in the genome. In REMOVER-PITCh targeting the *ARSB* locus, two multiplex vectors each expressing five sgRNAs and SpCas9 were constructed (no. 3, no. 4) (Fig. 2*b*, *c*). Of these sgRNAs, seven were designed to cleave the genomic intron for shredding, two were designed near the start and stop codons of the target gene, and one was designed to cleave the PITCh donor. The PITCh donor has a promoterless *ARSB* coding sequence (*ARSB* CDS)-T2A-NeoR cassette, which was flanked by 40-bp microhomologies (Fig. 2*b*). In addition, similar to the case of *GUSB*, silent mutations were introduced into the sgRNA target sequence on the *ARSB* CDS in the donor vector (Supplementary Fig. 4*a*). These vectors were co-transfected into the HCT116 cells and then drug-resistant cells were selected with G418. By the initial validation of PCR amplification to confirm the occurrence of replacement in cell populations, the objective bands at the 5′ and 3′ junctions and the full length of the cassette were detected, suggesting the presence of knock-in cells (Supplementary Fig. 4*b*, *c*). After single cell cloning, we performed PCR amplification of 5′ and 3′ junctions. The results indicated that 10 out of 16 clones were double positive for the 5′ and 3′ knock-in junctions (Fig. 2*d*). However, out-out PCR analysis resulted in all negative in these 10 clones (Fig. 2*e*, *f*), suggesting the imperfect knock-in. From these results, we hypothesized that the shredding of the intervening genomic region was not sufficient for the longer gene replacement, and further improvement was needed to solve this problem.

### Improvement of MMEJ knock-in efficiency using the LoAD system

**Figure 3.**
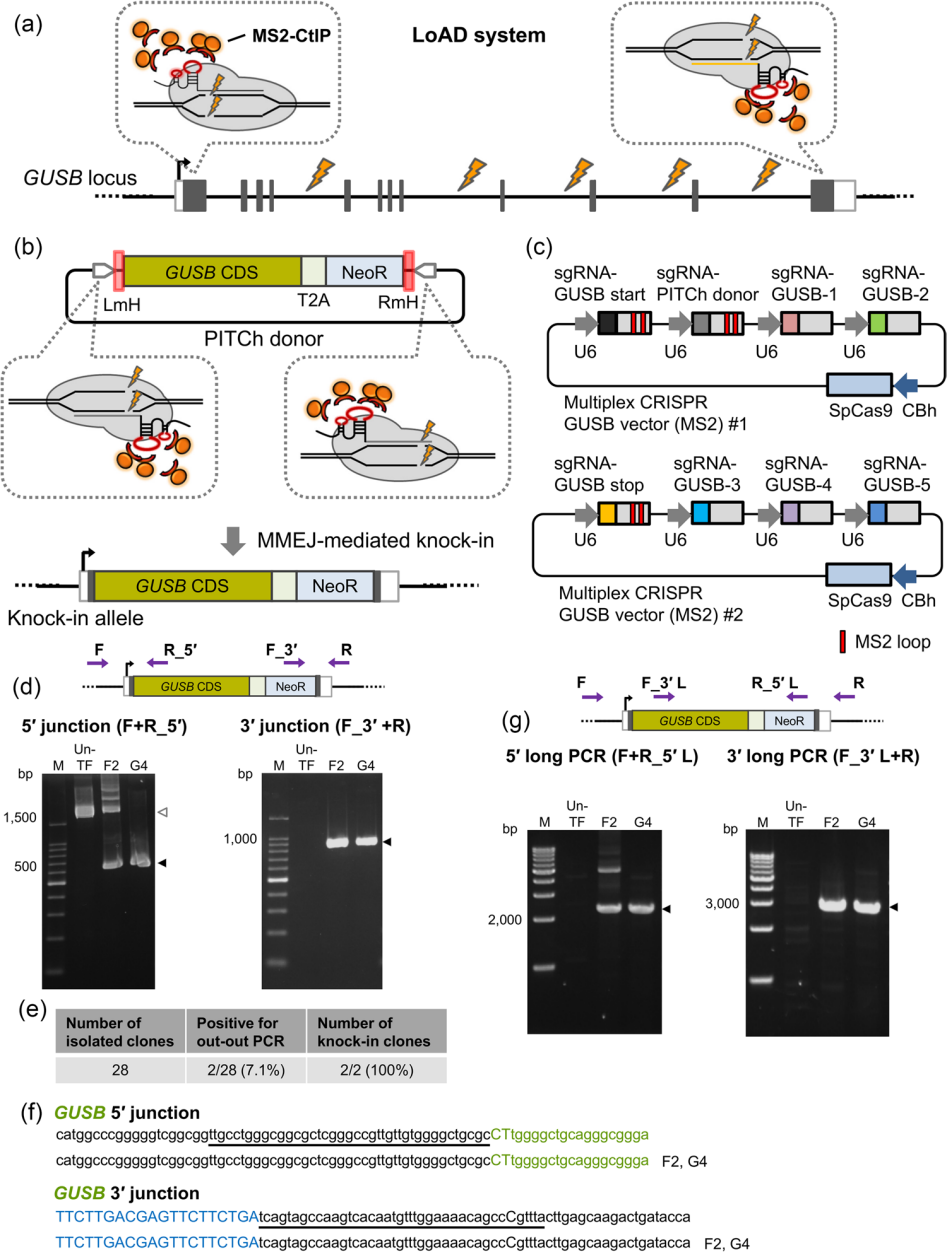
REMOVER-PITCh with the LoAD system at the *GUSB* locus. (*a*) Schematic illustration of REMOVER-PITCh with the LoAD system at the *GUSB* locus. *White* and *gray boxes* indicate 5′ and 3′ UTR, and exon, respectively. (*b*) Schematic illustration of the PITCh donor and the knock-in allele. The PITCh donor contains promoterless *GUSB* CDS-T2A-NeoR cassette flanked by 40-bp microhomologous sequences, which are indicated in *red boxes*. CDS, coding sequence. NeoR, neomycin resistance gene. LmH, left microhomologous sequence. RmH, right microhomologous sequence. (*c*) Schematic illustration of multiplex CRISPR vectors (MS2) for the *GUSB* locus. *Colored boxes* excluding SpCas9 indicate the target sequence of each sgRNA. *Light gray boxes* indicate consensus sequences of sgRNA. U6, human U6 promoter. CBh, chicken beta-actin hybrid promoter. SpCas9, *Streptococcus pyogenes* Cas9. (*d*, *g*) Confirmation of targeted replacement in the isolated clones by genomic PCR. *Purple arrows* on the schematic illustration of the knock-in allele indicate primers for PCR amplification. Genotyping was performed by junction PCR (F + R_5′ and F_3′ + R) (*d*) and long PCR (F + R_5′L and F_3′L + R) (*g*). The clone IDs are indicated at the top of each gel image. *Black* and *white triangles* indicate the amplicon sizes of knock-in alleles and non-knock-in alleles, respectively. M, ladder marker. Un-TF, untransfected cells. (*e*) Table summarizing the number of knock-in clones established. (*f*) Sequencing analysis of knock-in junctions in the isolated clones by Sanger sequencing. The intended knock-in sequence is shown at the top of each sequence. *Green* and *blue letters* indicate the coding sequences of *GUSB* and NeoR, respectively. *Underlines* indicate microhomologous sequences.

**Figure 4.**
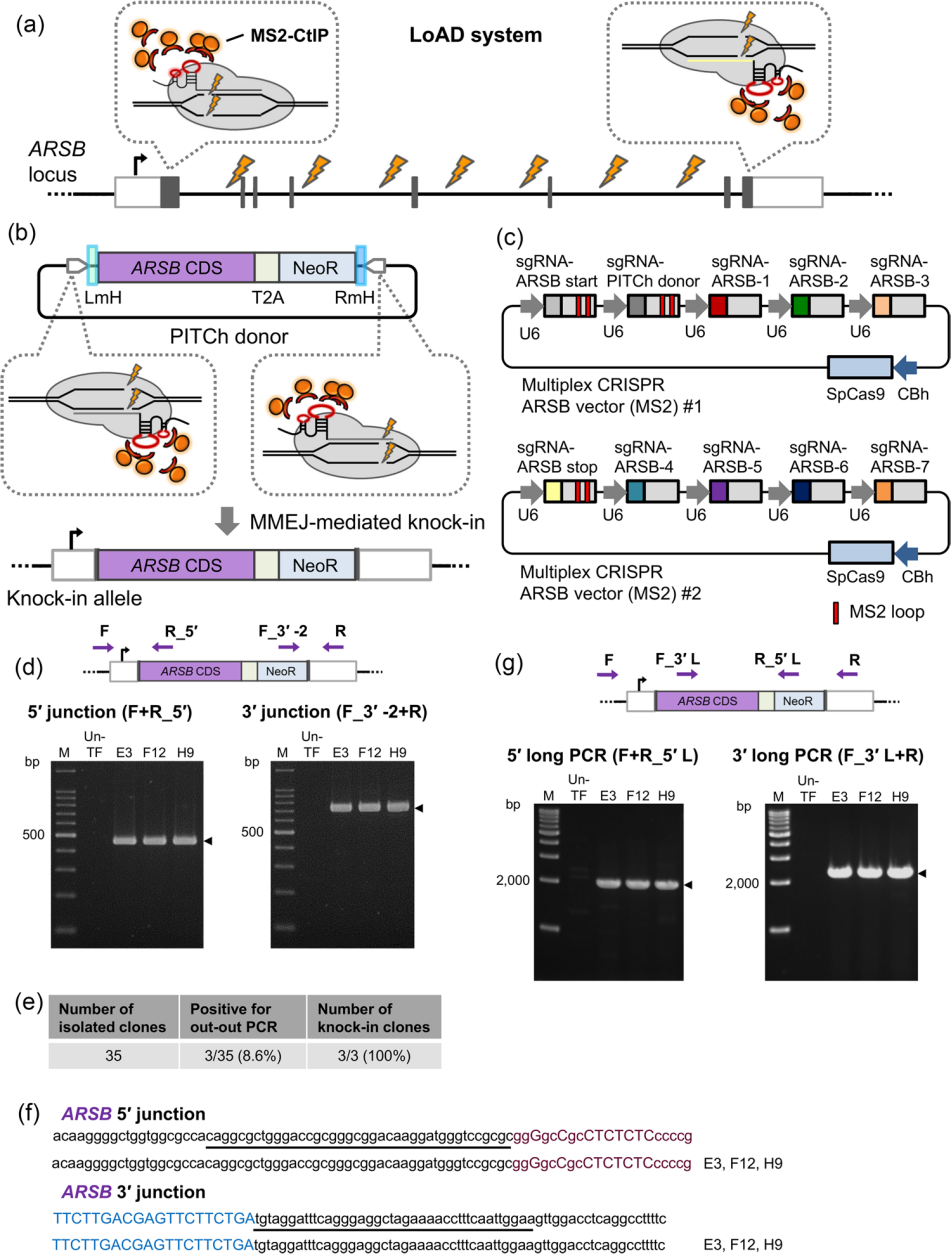
REMOVER-PITCh with the LoAD system at the *ARSB* locus. (*a*) Schematic illustration of REMOVER-PITCh with the LoAD system at the *ARSB* locus. *White* and *gray boxes* indicate 5′ and 3′ UTR, and exon, respectively. (*b*) Schematic illustration of the PITCh donor and the knock-in allele. The PITCh donor contains promoterless *ARSB* CDS-T2A-NeoR cassette flanked by 40-bp microhomologous sequences, which are indicated in *blue boxes*. CDS, coding sequence. NeoR, neomycin resistance gene. LmH, left microhomologous sequence. RmH, right microhomologous sequence. (*c*) Schematic illustration of multiplex CRISPR vectors (MS2) for the *ARSB* locus. Colored boxes excluding SpCas9 indicate the target sequence of each sgRNA. *Light gray boxes* indicate consensus sequences of sgRNA. U6, human U6 promoter. CBh, chicken beta-actin hybrid promoter. SpCas9, *Streptococcus pyogenes* Cas9. (*d*, *g*) Confirmation of targeted replacement in the isolated clones by genomic PCR. *Purple arrows* on the schematic illustration of the knock-in allele indicate primers for PCR amplification. Genotyping was performed by junction PCR (F + R_5′ and F_3′-2 + R) (*d*) and long PCR (F + R_5′L and F_3′L + R) (*g*). The clone IDs are indicated at the top of each gel image. *Black triangles* indicate the amplicon sizes of knock-in alleles. M, ladder marker. Un-TF, untransfected cells. (*e*) Table summarizing the number of knock-in clones established. (*f*) Sequencing analysis of knock-in junctions in the isolated clones by Sanger sequencing. The intended knock-in sequence is shown at the top of each sequence. *Purple* and *blue letters* indicate the coding sequences of *ARSB* and NeoR, respectively. Underlines indicate microhomologous sequences.

The local accumulation of DSB repair molecules (LoAD) system was previously developed as a method to improve the MMEJ-mediated knock-in efficiency (Nakade *et al*. 2018). In the LoAD system, sgRNA with MS2 loops and MMEJ repair-related factor, CtIP (Sfeir and Symington 2015; Anand *et al*. 2016), fused with MS2 coat protein (MS2-CtIP), are used to increase the MMEJ efficiency by accumulating CtIP around the DSB site. In this study, we attempted to improve the efficiency of gene replacement by REMOVER-PITCh at the *GUSB* and *ARSB* loci using the LoAD system (Figs. 3*a*, *b*, and 4*a*, *b*). For REMOVER-PITCh using the LoAD system, we modified the multiplex CRISPR vectors to express MS2 loop-containing sgRNAs responsible for MMEJ-mediated knock-in (Figs. 3*c*, and 4*c*).

First, we examined LoAD-assisted REMOVER-PITCh at the *GUSB* locus. The modified multiplex vectors, PITCh donor vector, and MS2-CtIP expression vector were co-transfected into HCT116 cells. A total of 28 clones were isolated by single cell cloning after drug selection. Genomic PCR and sequencing analyses of 5′ and 3′ junctions showed that 2/28 clones (7.1%) were positive for both junctions with precisely jointed sequences (Fig. 3*d*-*f*, Supplementary Fig. 5*a*, *b*). Furthermore, long PCR analysis suggested that these two clones possessed at least one perfect knock-in allele (Fig. 3*g*). We subsequently performed PCR amplification of the non-knock-in allele for these two clones. One out of two clones showed amplification of the 5′ region of the non-knock-in allele, and subsequent sequencing analysis revealed a single base insertion at the cleavage site (Supplementary Fig. 5*c*, *d*). Unexpectedly, some amplicons could be found even in the untransfected sample (Supplementary Fig. 5*a*, 6*a*, *d*). However, the knock-in-specific bands could not be produced from the same untransfected sample in the junction PCR analyses; therefore, we think that these bands do not affect the conclusion of our study. According to these results, we assumed that clone F2 was heterozygous and clone G4 was homozygous for the targeted replacement allele.

Next, we examined LoAD-assisted REMOVER-PITCh at the *ARSB* locus. Out-out PCR analysis of the isolated clones showed the presence of the replaced alleles in three out of 35 clones (8.6%), and junction PCR and sequencing analyses showed that these three clones had the correctly jointed junctions (Fig. 4*d*-*f*, Supplementary Fig. 6*a*, *b*). Additionally, long PCR analysis supported the presence of at least one perfect knock-in allele (Fig. 4*g*). As a result of non-knock-in allele-specific PCR analysis (Supplementary Fig. 6*c*), the amplified bands appeared in all three clones (Supplementary Fig. 6*c*, *d*). Sequencing analysis of the PCR products showed a single nucleotide insertion at the cleavage site in clones E3 and F12, and a two-base deletion in clone H9 at the 5′ side. At the 3′ side, a partial donor sequence (2A-NeoR-RmH) was incorporated in all clones (Supplementary Fig. 6*e*). These results indicated that the three clones were heterozygous for the replacement.

Taken together, we found that the LoAD system could improve the efficiency of targeted replacement by REMOVER-PITCh. With the assistance of the LoAD system, we achieved MMEJ-dependent gene replacement at a large 200-kb genomic region in the *ARSB* locus. On the other hand, the efficiency of targeted replacement using the LoAD system was comparable with that without the LoAD system at the *GUSB* locus. At the *GUSB* locus, the imperfect knock-in was not observed in the established clones even without the LoAD system; therefore, the biasing effect of MMEJ repair by the LoAD system might not be necessary at this locus.

### Off-target analysis

Since up to 10 sgRNAs were co-expressed within the cells for REMOVER-PITCh, the possibility of off-target effect is a major concern of our system. Therefore, we checked the potential off-target sites in the knock-in clones by Cel-I assay and direct sequencing. For each knock-in locus, the top seven candidate sites were selected using the COSMID web tool (Cradick *et al*. 2014). In each clone, the seven candidate sites were PCR-amplified, and evaluated the presence or non-presence of mutations at those sites using the GeneArt Genomic Cleavage Detection Kit. The results showed that no obvious mutations were introduced at all seven candidate sites in each clone (Supplementary Fig. 7*a*, *b*). In addition, no off-target mutations were detected by direct sequencing of the PCR products (Supplementary Fig. 7*c*, *d*).

## Conclusion and future perspectives

In this study, we designed a REMOVER-PITCh system enabling gene replacement in a large genome region and then demonstrated the utility of this system at two genomic loci in human cultured cells. REMOVER-PITCh is a method of replacing the target genomic region with a target cassette by inducing multiple cleavages at the target region and a donor vector using multiple sgRNAs. We achieved targeted replacement with 6% efficiency using this system at around 20-kb region of the *GUSB* locus. Furthermore, to increase the replacement efficiency at a larger region, we utilized the LoAD system for REMOVER-PITCh, achieving the replacement efficiency of 8.6% at around 200-kb region of the *ARSB* locus.

Several similar studies have been reported previously. Danner *et al*. (2021) showed that a genome region of about 400 bp at three loci could be replaced by a 1.3-kb reporter cassette with 16–54% efficiency in human cultured cells using dual-sgRNAs and NHEJ. Katayama *et al*. (2019) also succeeded in replacing a genomic region of about 3–5 kb with a reporter cassette of about 400 bp via MMEJ at two loci in mouse cells with about 20% efficiency. However, only partial gene regions within 10 kb were removed in these reports. Zhang *et al*. (2015) and Ohno *et al*. (2022) showed HR-mediated gene replacement in mES cells and HCT116 cells, respectively, for longer gene regions up to ~ 100 kb and longer, although the applicability of these systems in cells and animals with low HR activity is unclear. REMOVER-PITCh established in this study showed the potential for efficient gene replacement in large gene regions without depending on HR.

In our examination, targeted replacement by REMOVER-PITCh was performed using wild-type SpCas9 and a particular number of sgRNAs as a demonstration of the system. To make this system more efficient and safer, some optimization would be needed. For example, the risk of off-target cleavages can reasonably be reduced by using highly specific Cas9 variants or truncated gRNA (Fu *et al*. 2014; Kleinstiver *et al*. 2016; Slaymaker *et al*. 2016; Vakulskas *et al*. 2018). In addition, although we showed the non-existence of off-target mutations at the top-ranked candidate sites, deeper analysis throughout the genome will be needed especially for medical applications. The evaluation of our system in different cell lines and organisms is also a future challenge.

As a future perspective, the REMOVER-PITCh system, an attractive option for a large gene replacement, is expected to facilitate the creation of humanized mice and disease models and help elucidate pathological mechanisms. Also in the field of therapeutics, the REMOVER-PITCh system is expected to be utilized in the removal of pathogenically mutated genes and the insertion of therapeutic genes via the replacement of large genomic regions.

### Supplementary Information

Below is the link to the electronic supplementary material.

Supplementary file1 (DOCX 7523 KB)

## Data Availability

The sequence information of the oligonucleotides used for the primers and the templates of the sgRNAs, and the donor constructs are provided in Supplementary Table 1–3 and Supplementary Sequences.
