# Supplementary material for: REMOVER-PITCh: microhomology-assisted long-range gene replacement with highly multiplexed CRISPR-Cas9
Source: In Vitro Cell Dev Biol Anim. 2024 Feb 9;60(7):697–707. doi: 10.1007/s11626-024-00850-1 (PMC11297102; doi:10.1007/s11626-024-00850-1)
Supplement: Supplementary file 1 — Supplementary file1 (DOCX 7523 KB) [file 11626_2024_850_MOESM1_ESM.docx]

**Supplementary information**

**REMOVER-PITCh: microhomology-assisted long-range gene replacement with highly multiplexed CRISPR-Cas9**

Shu Matsuzaki^1, 2^, Tetsushi Sakuma^1, *^, and Takashi Yamamoto^1^

^1^Division of Integrated Sciences for Life, Graduate School of Integrated Sciences for Life, Hiroshima University, 1-3-1 Kagamiyama, Higashi-Hiroshima, Hiroshima 739-8526, Japan

^2^Drug Discovery Laboratory, Wakunaga Pharmaceutical Co., Ltd., 1624 Shimokotachi, Koda-cho, Akitakata-shi, Hiroshima 739-1195, Japan

*Correspondence should be addressed to T.S.

Division of Integrated Sciences for Life,

Graduate School of Integrated Sciences for Life, Hiroshima University,

1-3-1 Kagamiyama, Higashi-Hiroshima, Hiroshima 739-8526, Japan

E-mail: tetsushi-sakuma@hiroshima-u.ac.jp

Tel.: +81-82-424-6292

**This file includes:**

**Supplementary Figure 1–7**

**Supplementary Table 1–3**

**Supplementary Sequences**

**Supplementary Figures**

**
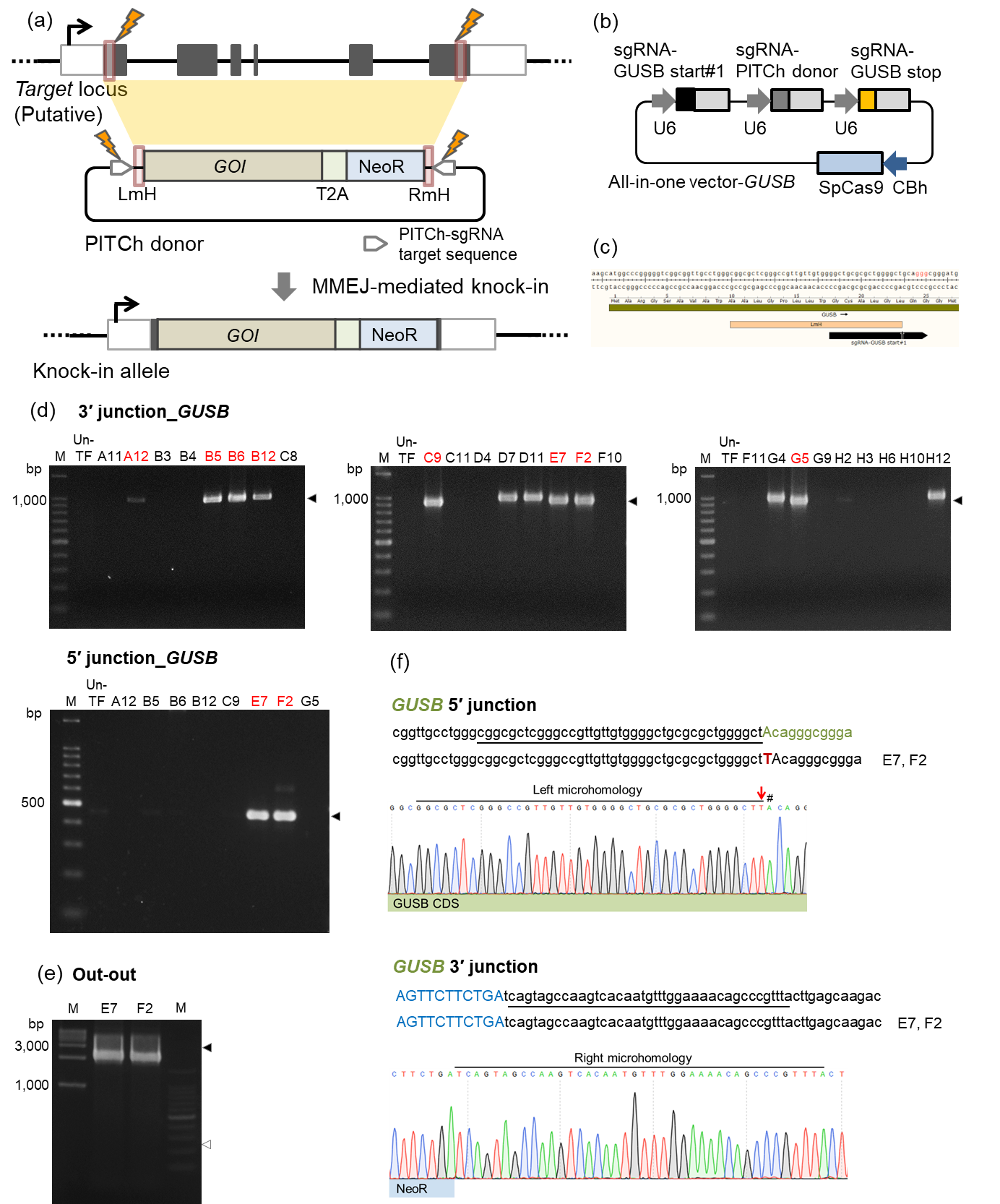
**

**
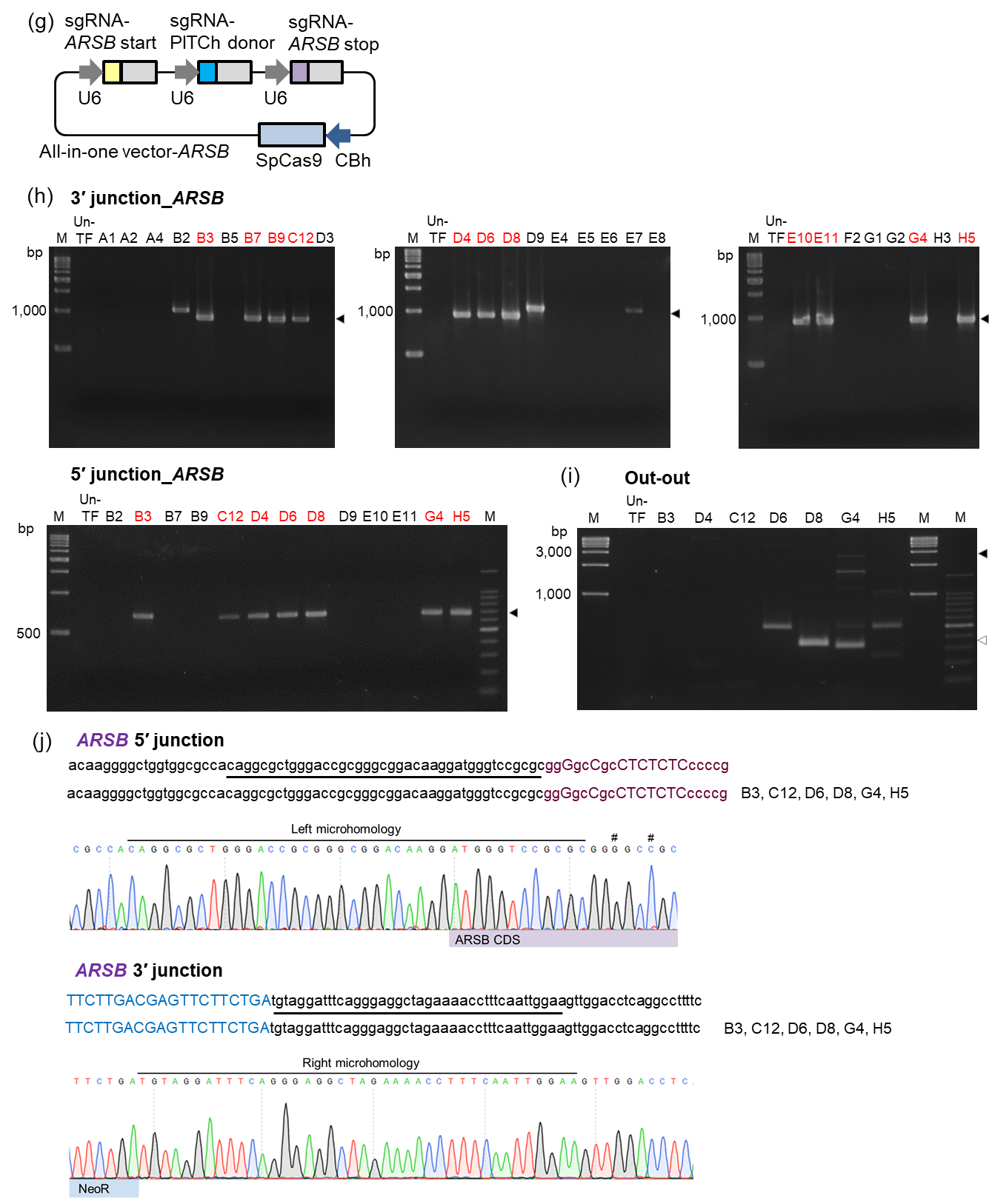
**

**
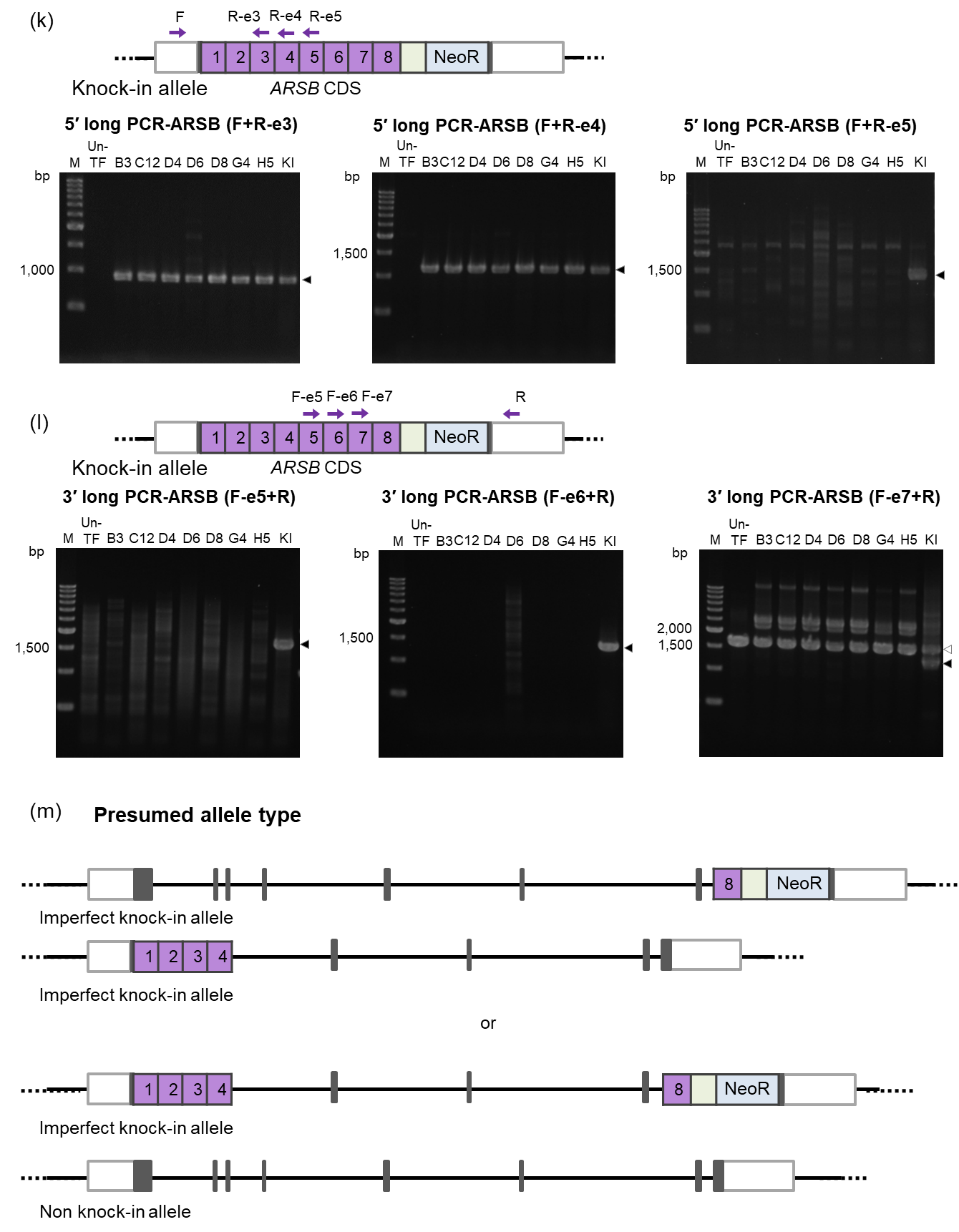
**

**Supplementary Figure 1. Trial of standard PITCh-mediated gene replacement at the *GUSB* and *ARSB* loci.**

**a** Schematic illustration of standard PITCh-mediated gene replacement. Putative genomic context, the PITCh donor, and the intended knock-in allele are shown. White and gray boxes indicate 5' and 3' UTR, and exon, respectively. The PITCh donor contains a *GOI*-T2A-NeoR cassette flanked by 40-bp microhomologous sequences (LmH, RmH), which are indicated in red boxes. GOI, gene of interest. NeoR, neomycin resistance gene. LmH, left microhomologous sequence. RmH, right microhomologous sequence. **b, g** Schematic illustration of all-in-one CRISPR vectors targeting the *GUSB* locus (**b**) and the *ARSB* locus (**g**). Colored boxes excluding SpCas9 indicate the target sequence of each sgRNA. Light gray boxes indicate consensus scaffold sequences of sgRNA. U6, human U6 promoter. CBh, chicken beta-actin hybrid promoter. SpCas9, *Streptococcus pyogenes* Cas9. **c** The target sequence of sgRNA-GUSB start#1 and the left microhomologous sequence (LmH). The PAM sequence is highlighted in red letters. Other target sequences, sgRNA-GUSB stop, sgRNA-ARSB start, and sgRNA-ARSB stop, are shown in Fig. 1a and 2a. **d, e, h, i** Genotyping analysis by junction PCR and out-out PCR in the isolated clones for the *GUSB* locus (**d**, **e**) and the *ARSB* locus (**h**, **i**). The clone IDs are indicated at the top of each gel image. The clones showing the intended amplicon sizes are highlighted in red. Black and white triangles indicate the appropriate sizes of each amplicon from the intended knock-in allele and the chromosomally deleted allele, respectively. M, DNA ladder marker. Un-TF, untransfected. **f, j** Sequencing analysis of knock-in junctions. The intended knock-in sequences are shown at the top of each sequence. Underlines indicate microhomologous sequences. The red uppercase letter and red arrow indicate the base and the position of a single base insertion, respectively. #, silent mutation. **k**, **l** Long PCR analysis using primers designed at each exon. Purple arrows on the illustration of the intended knock-in allele indicate primers for PCR amplification. Black and white triangles indicate the appropriate sizes of each amplicon from the intended knock-in allele and non-knock-in allele, respectively. KI, Drug-selected cell populations. **m** Schematic illustration of the presumed imperfect knock-in alleles.


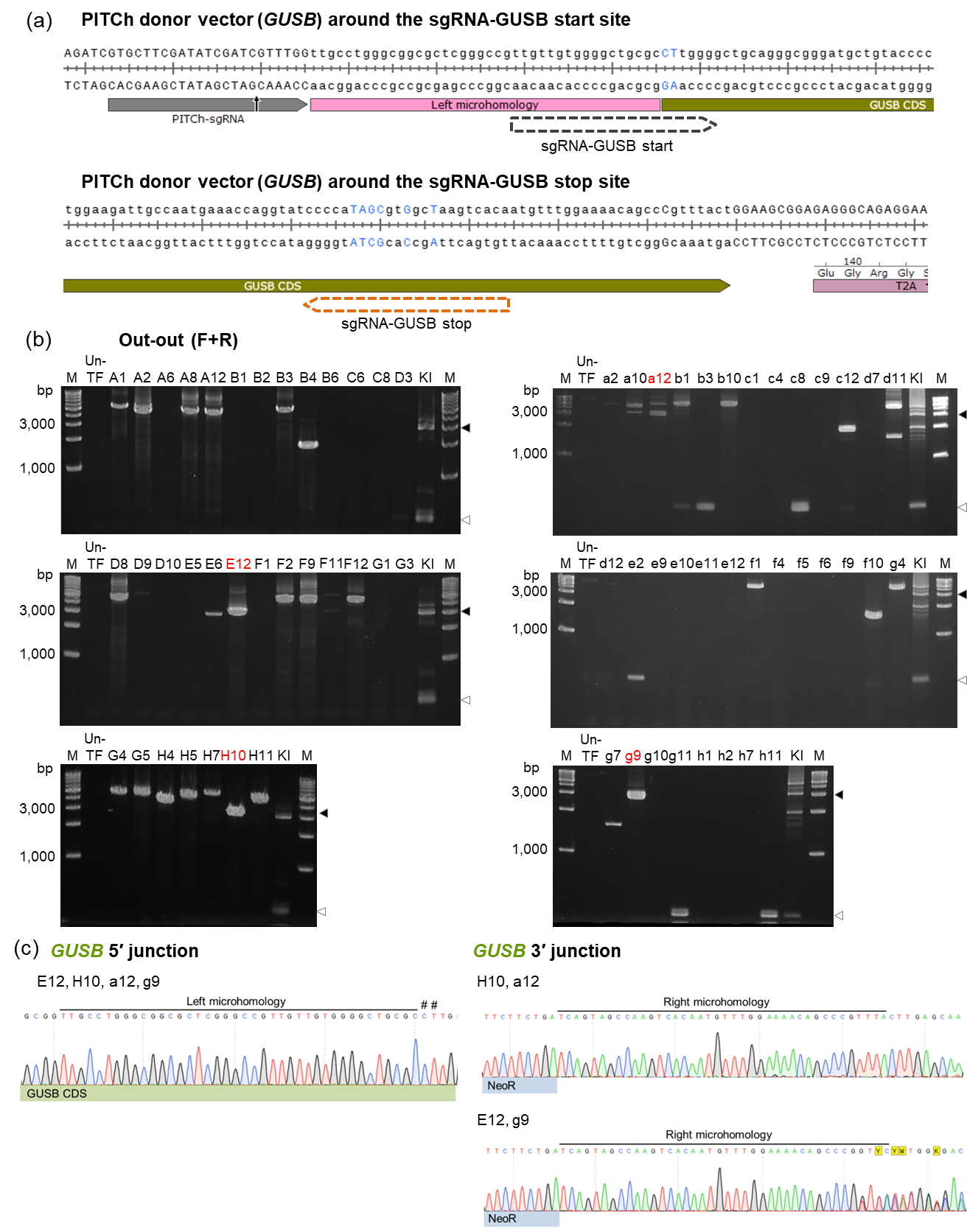


**Supplementary Figure 2. Design, genotyping, and sequencing of REMOVER-PITCh at the *GUSB* locus, related to Fig. 1.**

**a** Silent mutations were introduced into the sgRNA-GUSB start and sgRNA-GUSB stop target sequences on the PITCh donor. Silent mutations are highlighted in blue letters. **b** Out-out PCR analysis for the isolated clones. The clone IDs are indicated at the top of each gel image. The clones showing the intended amplicon sizes were highlighted in red. Black and white triangles indicate the amplificon sizes of the intended knock-in allele and the chromosomally deleted allele, respectively. M, DNA ladder marker. Un-TF, untransfected. KI, Drug-selected cell populations. **c** Sequencing analysis of knock-in junctions in the four clones. Underlines indicate microhomologous sequences. #, silent mutations.

**
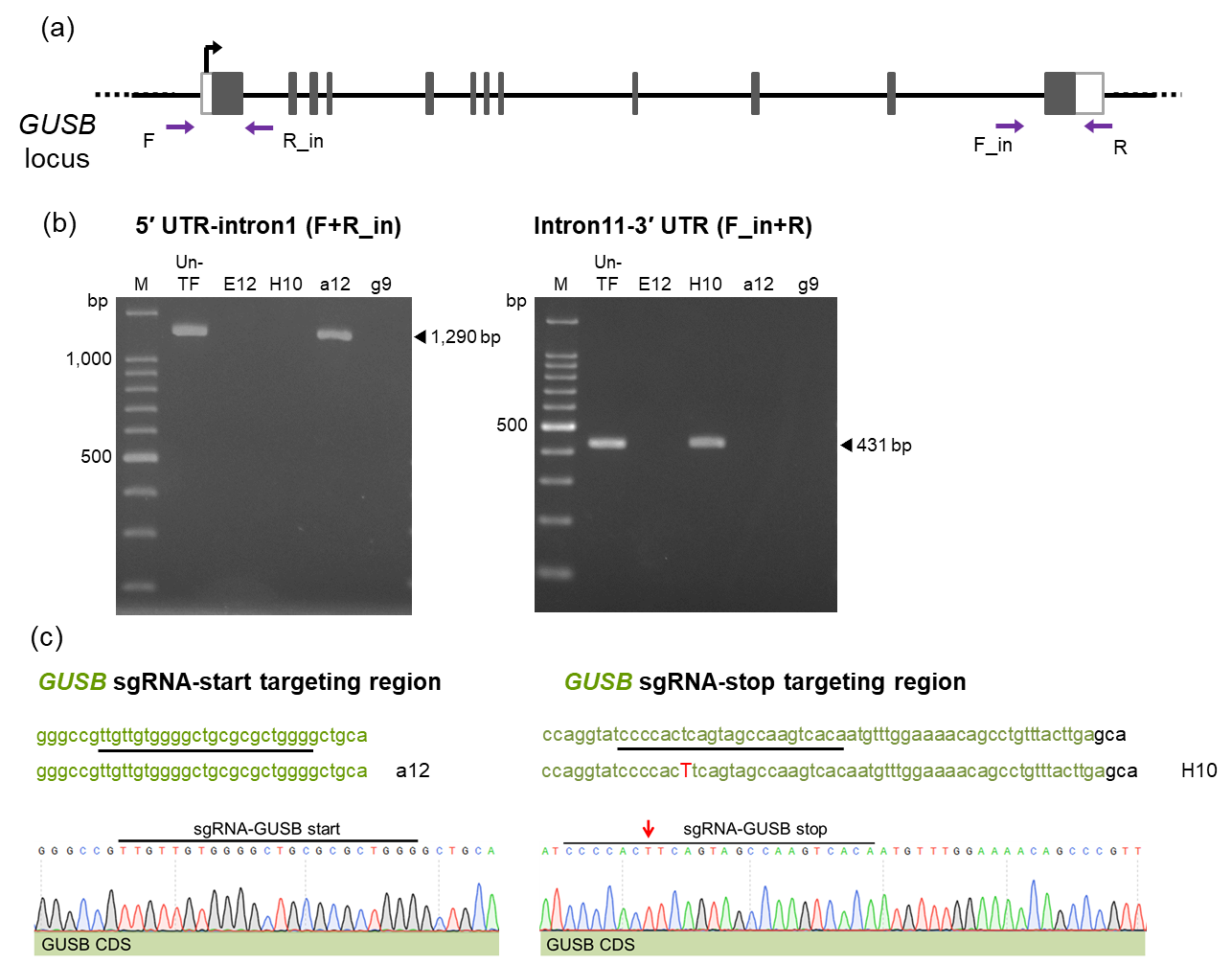
**

**Supplementary Figure 3. Examination of non-knock-in alleles in the knock-in clones at the *GUSB* locus, related to Fig. 1.**

**a** Schematic illustration of wild-type allele at the *GUSB* locus. Purple arrows indicate primers on UTR and intron. **b** Non-knock-in allele-specific genomic PCR analysis. The clone IDs are indicated at the top of each gel image. Black triangles indicate the appropriate sizes of each amplicon from the non-knock-in allele. M, DNA ladder marker. Un-TF, untransfected. **c** Sequencing analysis of the amplicons derived from clones a12 and H10. The sequences of wild-type alleles are shown at the top of each sequence. Underlines indicate sgRNA target sequences. Insertion is highlighted in a red letter. Green letters indicate the *GUSB* coding sequence. The red arrow indicates the position of the insertion.


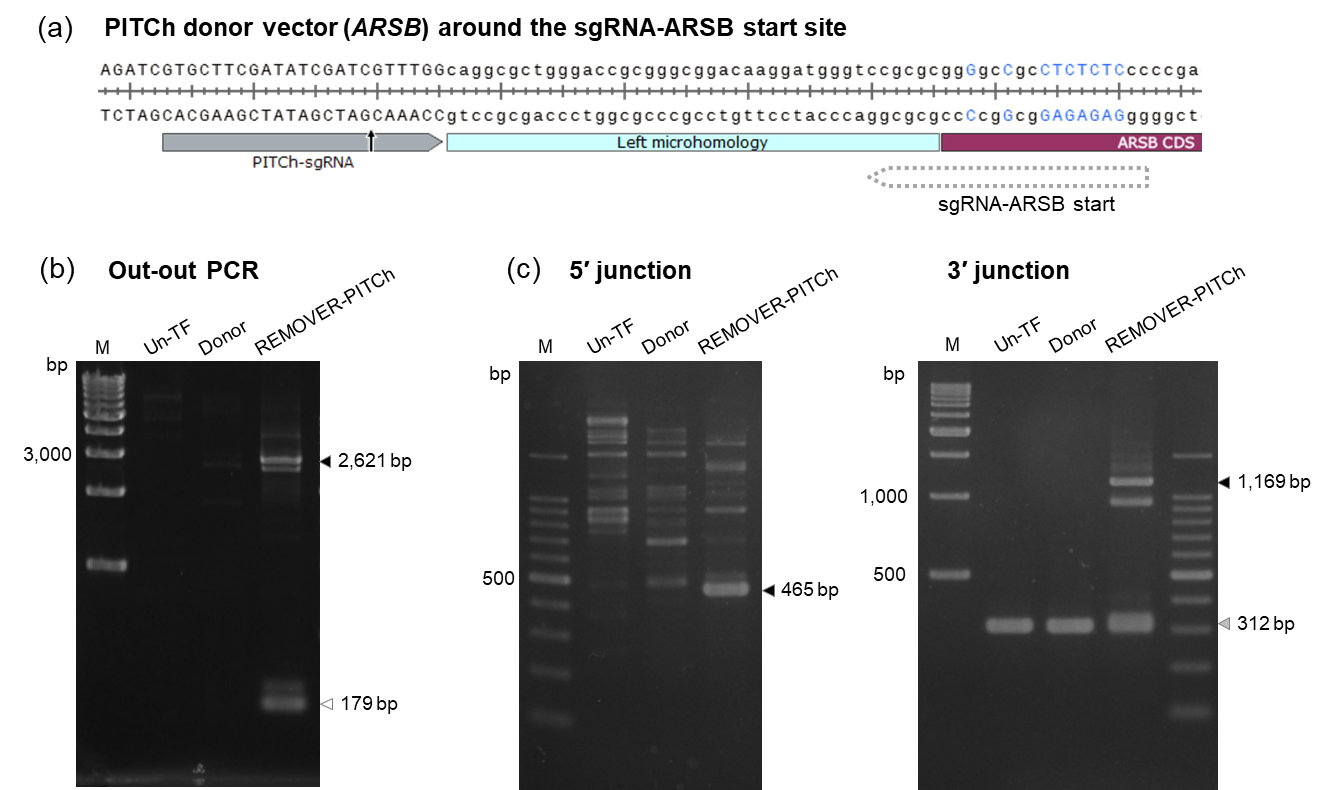


**Supplementary Figure 4. Design and initial validation of REMOVER-PITCh at the *ARSB* locus, related to Fig. 2.**

**a** Silent mutations were introduced into the sgRNA-ARSB start target sequence on the PITCh donor. Silent mutations are highlighted in blue letters. **b, c** Initial validation by population analysis of replacement with out-out PCR (**b**) and junction PCR (**c**). Black, white, and gray triangles indicate the amplificon sizes of the intended knock-in allele, the chromosomally deleted allele, and the wild-type allele, respectively. M, DNA ladder marker. Un-TF, untransfected.

**
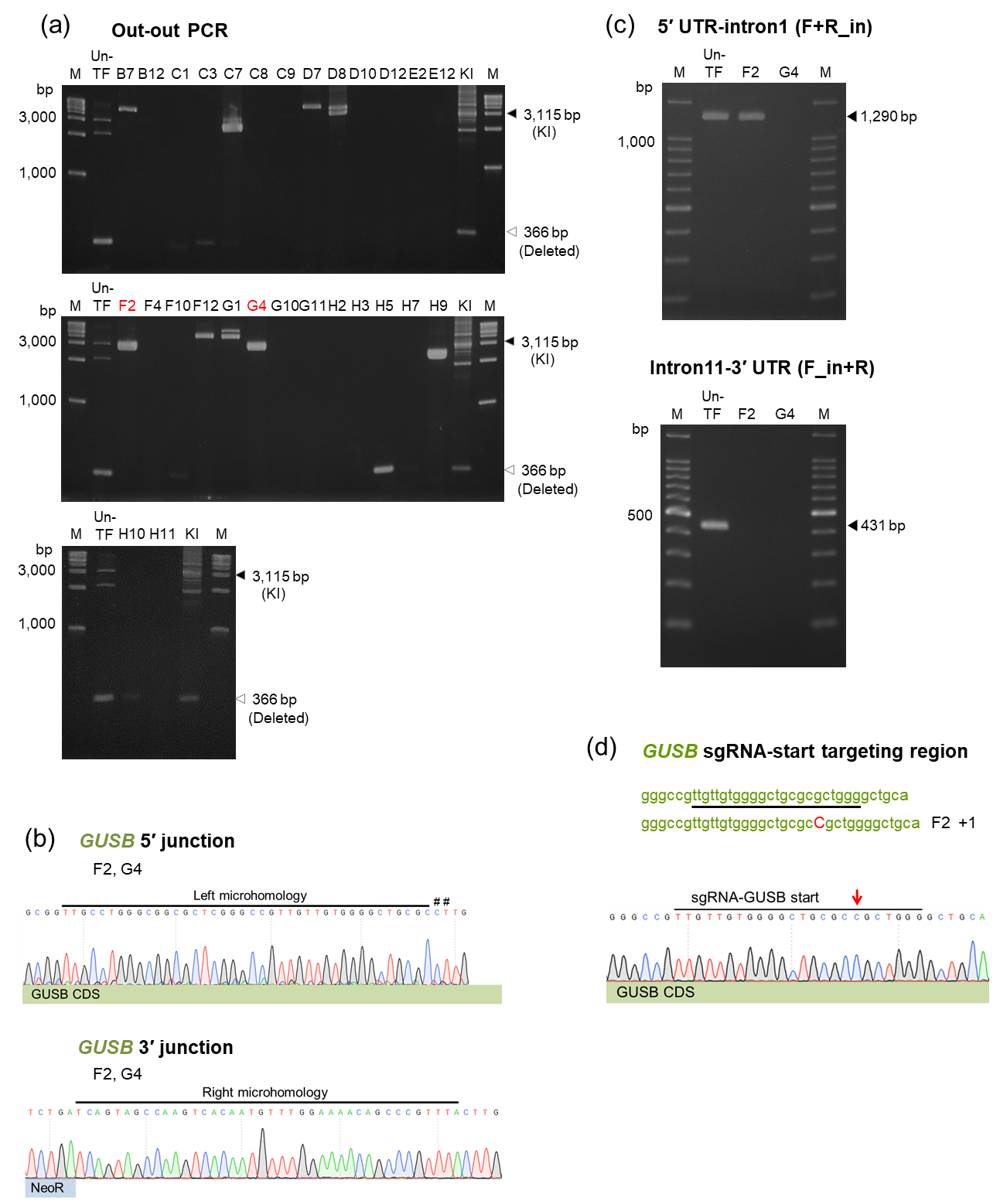
**

**Supplementary Figure 5. Genotyping and sequencing of LoAD-assisted REMOVER-PITCh at the *GUSB* locus, related to Fig. 3.**

**a** Out-out PCR for the isolated clones. The clone IDs are indicated at the top of each gel image. The clones showing the intended amplicon sizes were highlighted in red. Black and white triangles indicate the amplificon sizes of the intended knock-in allele and the chromosomally deleted allele, respectively. M, DNA ladder marker. Un-TF, untransfected. KI, Drug-selected cell populations. **b** Sequencing analysis of 5' and 3' junctions for the knock-in clones. #, silent mutations. **c** Non-knock-in allele-specific genomic PCR analysis. **d** Sequencing analysis of the amplicon derived from clone F2. The sequence of wild-type alleles is shown at the top. The sgRNA target sequence is underlined. Insertion is highlighted in a red letter. Green letters indicate the *GUSB* coding sequence. The red arrow indicates the position of the insertion.

**
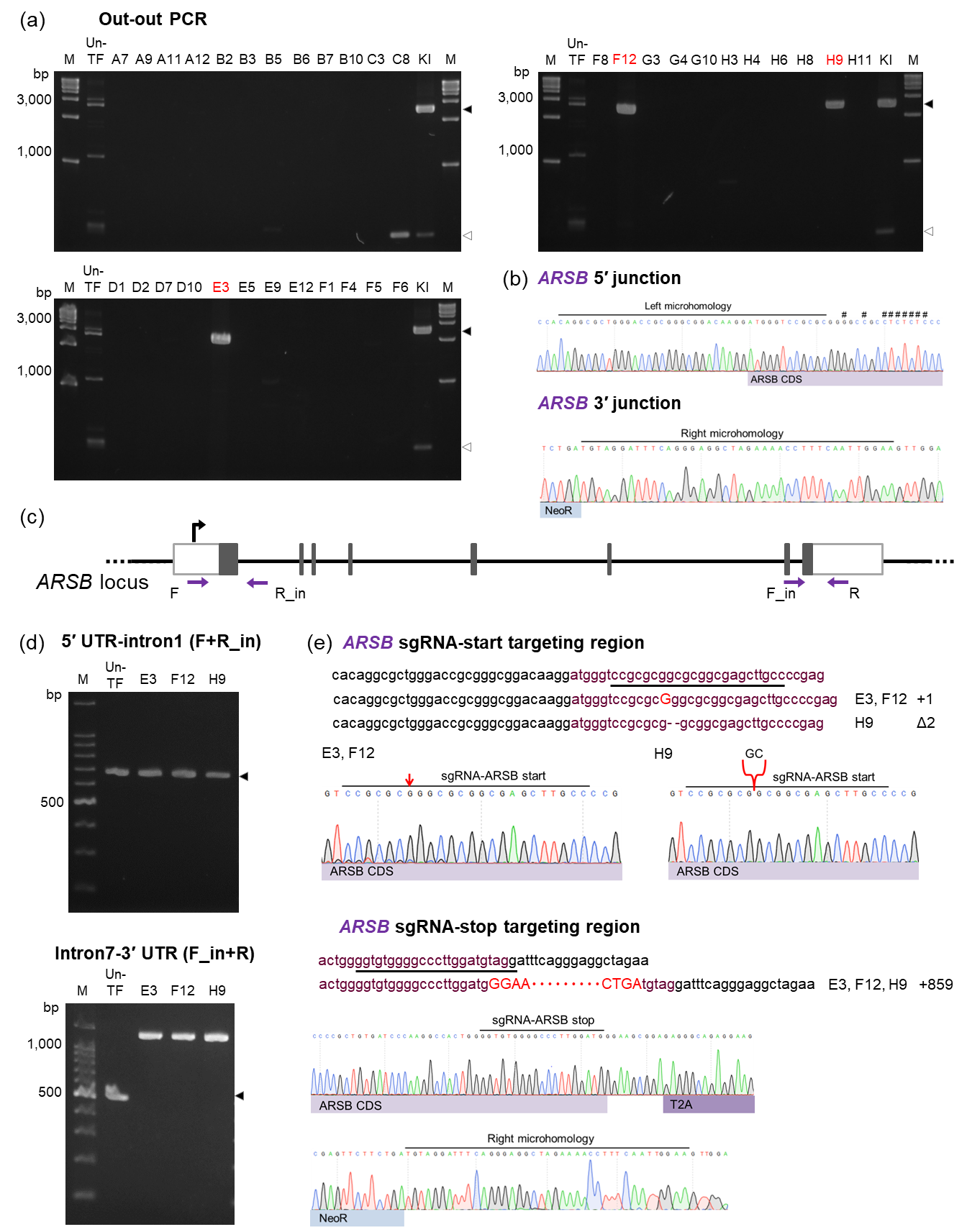
**

**Supplementary Figure 6. Genotyping and sequencing of LoAD-assisted REMOVER-PITCh at the *ARSB* locus, related to Fig. 4.**

**a** Out-out PCR for the isolated clones. The clone IDs are indicated at the top of each gel image. The clones showing the intended amplicon sizes were highlighted in red. Black and white triangles indicate the amplificon sizes of the intended knock-in allele and the chromosomally deleted allele, respectively. M, DNA ladder marker. Un-TF, untransfected. KI, Drug-selected cell populations. **b** Sequencing analysis of 5' and 3' junctions for the knock-in clones. #, silent mutations. **c** Schematic illustration of wild-type allele at the *GUSB* locus. Purple arrows indicate primers on UTR and intron. **d** Non-knock-in allele-specific genomic PCR analysis. **e** Sequencing analysis of the amplicons derived from clones E3, F12, and H9. The sequences of wild-type alleles are shown at the top of each sequence. The sgRNA target sequences are underlined. Insertions are highlighted in red letters. Purple letters indicate the *ARSB* coding sequence. The red arrow indicates the position of the insertion. Hyphens indicate deletions.

**
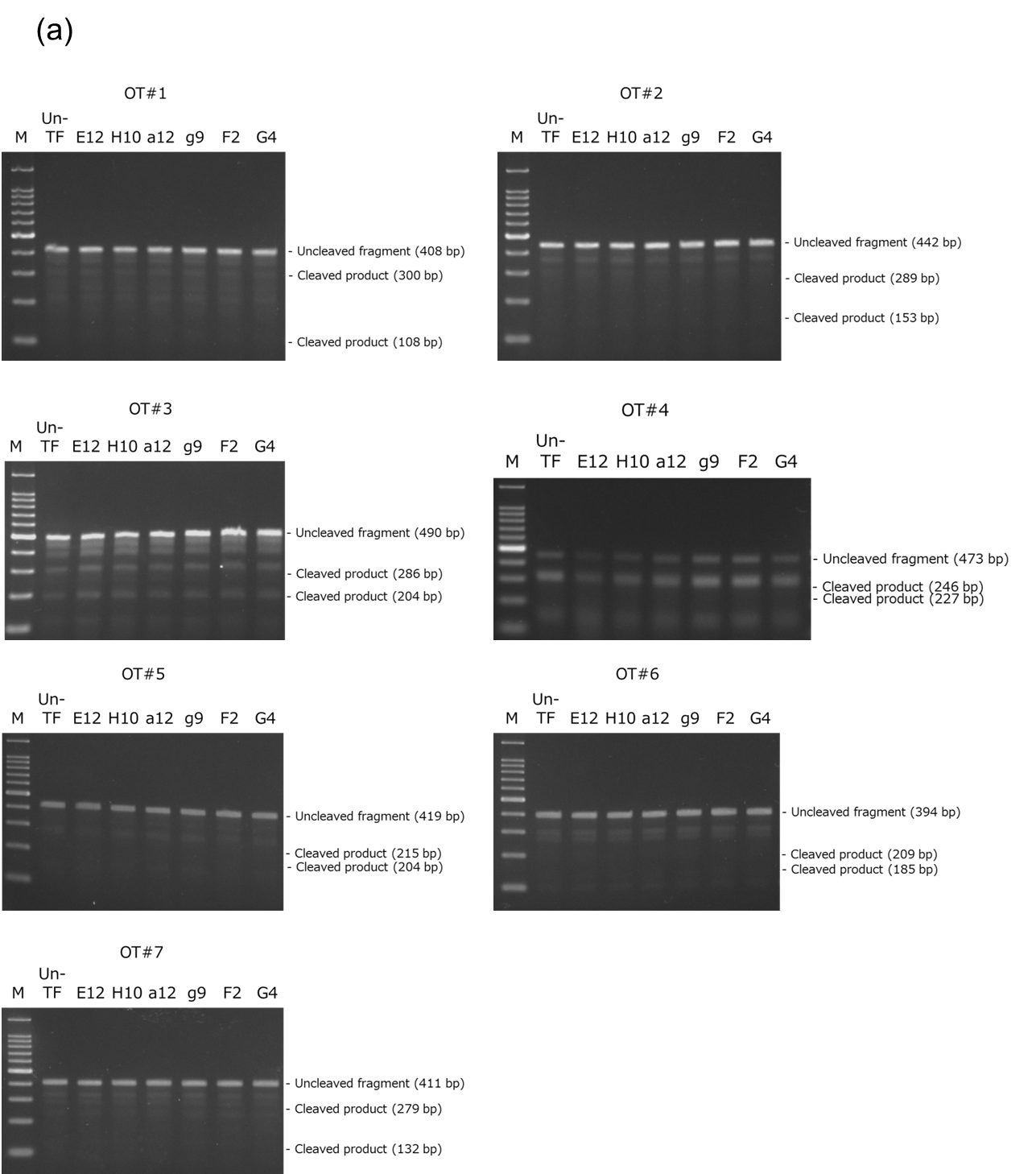
**

**
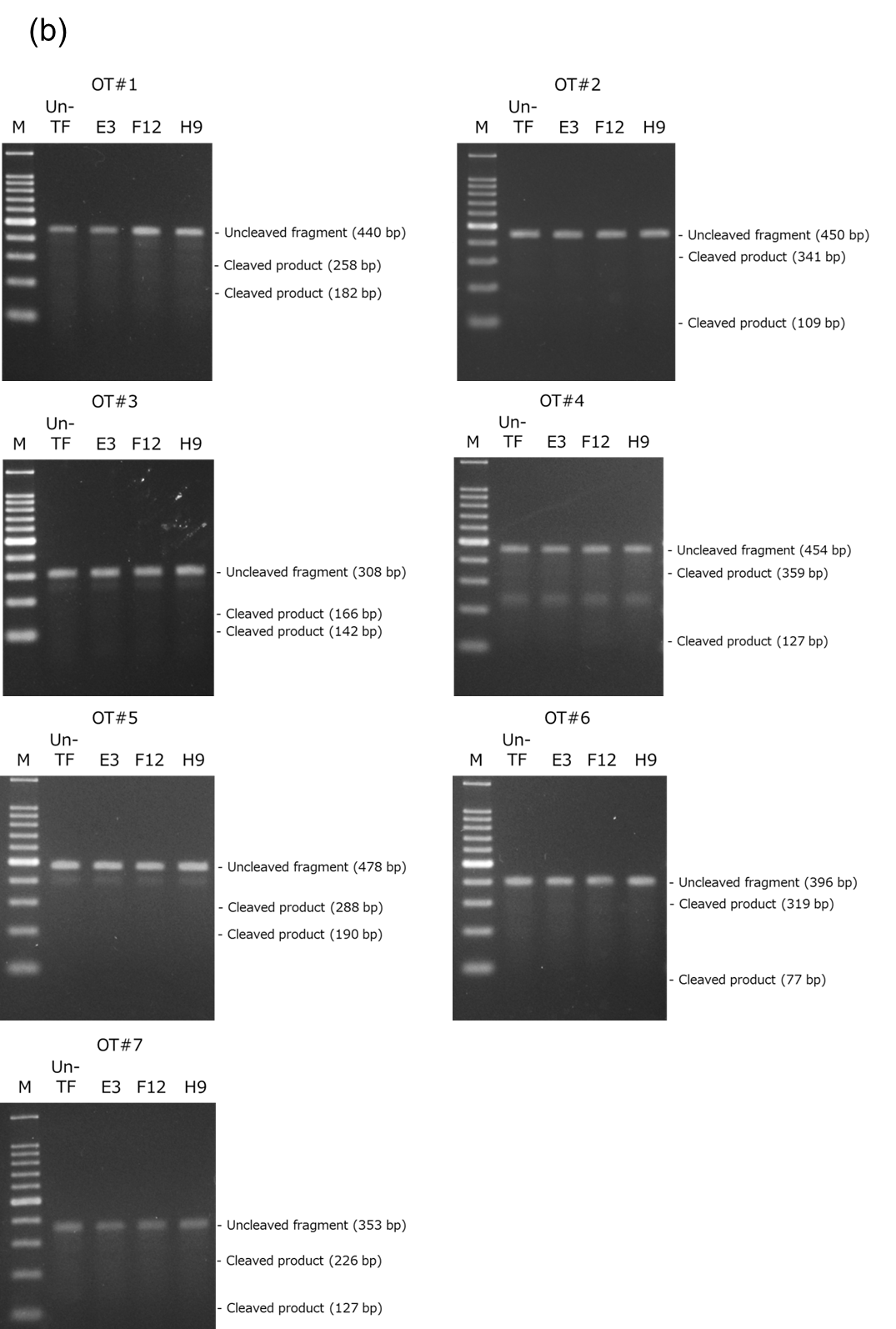
**

**
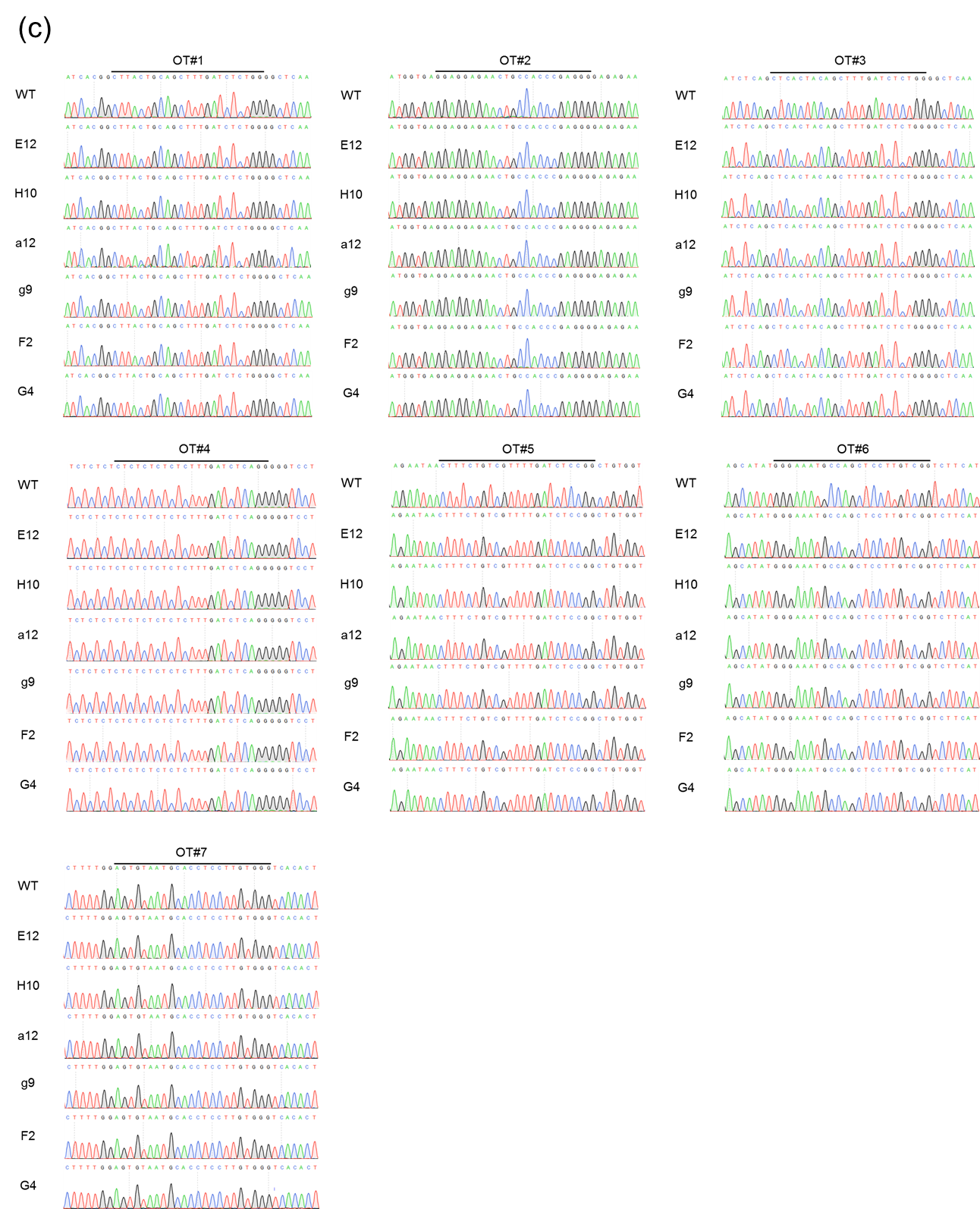
**

**
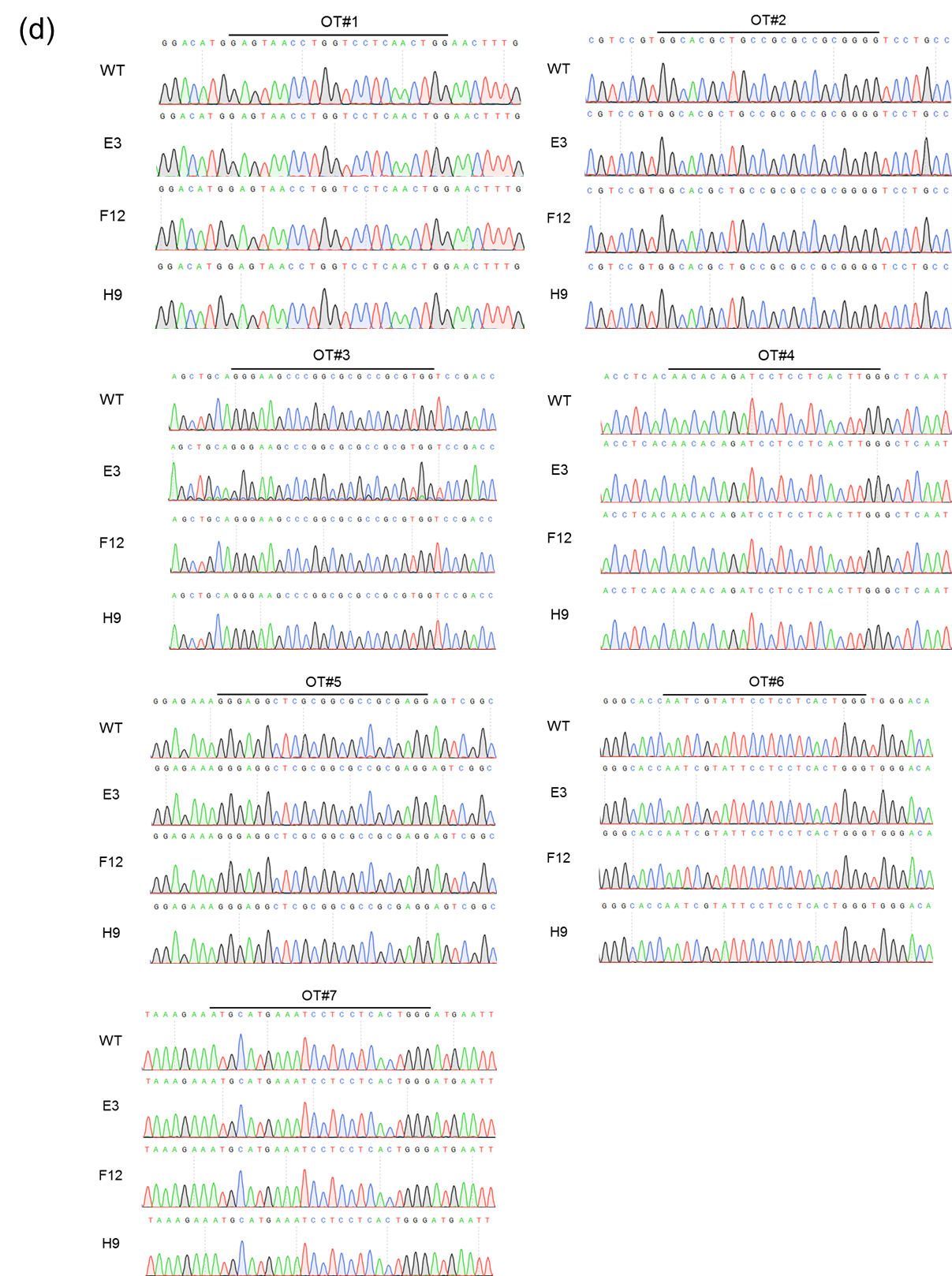
**

**Supplementary Figure 7. Off-target analysis in knock-in clones.** Confirmation of the existence or the non-existence of off-target mutations in knock-in clones of the *GUSB* locus (**a, c**) and the *ARSB* locus (**b, d**) by Cel-I assay (**a, b**) and sequencing analysis (**c, d**). The top seven candidate sites (OT#1–#7) were examined. The clone IDs are indicated at the top (**a, b**) or left (**c, d**) of each panel. The sgRNA target sequence is underlined. M, DNA ladder marker. Un-TF, untransfected.

**Supplementary Tables**

**Supplementary Table 1**

Oligonucleotides for sgRNAs of PITCh-replacement

| **Locus** | **Region** |  | **Sequence (5′ to 3′)** |
| --- | --- | --- | --- |
| *GUSB* | Exon 1 | Sense | CACCGGCTGCGCGCTGGGGCTGCA |
|  |  | Antisense | AAACTGCAGCCCCAGCGCGCAGCC |
|  | Exon 12 | Sense | CACCGTGTGACTTGGCTACTGAGTG |
|  |  | Antisense | AAACCACTCAGTAGCCAAGTCACAC |
| *ARSB* | Exon 1 | Sense | CACCGGCAAGCTCGCCGCGCCGCG |
|  |  | Antisense | AAACCGCGGCGCGGCGAGCTTGCC |
|  | Exon 8 | Sense | CACCGGTGTGGGGCCCTTGGATGT |
|  |  | Antisense | AAACACATCCAAGGGCCCCACACC |

| **Locus** | **Region** |  | **Sequence (5′ to 3′)** |
| --- | --- | --- | --- |
| PITCh-donor | PITCh-sgRNA | Sense | GTGCTTCGATATCGATCGTTTGG |
|  |  | Antisense | CCAAACGATCGATATCGAAGCAC |

Oligonucleotides for sgRNAs of REMOVER-PITCh

| **Locus** | **Region** |  | **Sequence (5′ to 3′)** |
| --- | --- | --- | --- |
| *GUSB* | Exon 1 | Sense | CACCGTTGTTGTGGGGCTGCGCGCT |
|  |  | Antisense | AAACAGCGCGCAGCCCCACAACAAC |
|  | Exon 12 | Sense | CACCGTGTGACTTGGCTACTGAGTG |
|  |  | Antisense | AAACCACTCAGTAGCCAAGTCACAC |
|  | Intron 4 | Sense | CACCGAGATTATTGACAGCCGTTGT |
|  |  | Antisense | AAACACAACGGCTGTCAATAATCTC |
|  | Intron 8 | Sense | CACCGTCATTGCTCTGCCTATCCGA |
|  |  | Antisense | AAACTCGGATAGGCAGAGCAATGAC |
|  | Intron 9 | Sense | CACCGGAGAAGCGCTGCCACCCGA |
|  |  | Antisense | AAACTCGGGTGGCAGCGCTTCTCC |
|  | Intron 10 | Sense | CACCGCTCACTGTCGCTTTGATCTC |
|  |  | Antisense | AAACGAGATCAAAGCGACAGTGAGC |
|  | Intron 11 | Sense | CACCGGATAATGCCACCTCCTTGT |
|  |  | Antisense | AAACACAAGGAGGTGGCATTATCC |

| **Locus** | **Region** |  | **Sequence (5′ to 3′)** |
| --- | --- | --- | --- |
| *ARSB* | Exon 1 | Sense | CACCGGCAAGCTCGCCGCGCCGCG |
|  |  | Antisense | AAACCGCGGCGCGGCGAGCTTGCC |
|  | Exon 8 | Sense | CACCGGTGTGGGGCCCTTGGATGT |
|  |  | Antisense | AAACACATCCAAGGGCCCCACACC |
|  | Intron 1 | Sense | CACCGAAGTAACTCTGGTCCTCAAC |
|  |  | Antisense | AAACGTTGAGGACCAGAGTTACTTC |
|  | Intron 4_1 | Sense | CACCGATACATATATCCTCCTCACT |
|  |  | Antisense | AAACAGTGAGGAGGATATATGTATC |
|  | Intron 4_2 | Sense | CACCGTGGGTTCATTACATTGAGAG |
|  |  | Antisense | AAACCTCTCAATGTAATGAACCCAC |
|  | Intron 5_1 | Sense | CACCGCATTAGGCAAGTTCGGAAT |
|  |  | Antisense | AAACATTCCGAACTTGCCTAATGC |
|  | Intron 5_2 | Sense | CACCGAAGGAGTTTCACCGGGATAG |
|  |  | Antisense | AAACCTATCCCGGTGAAACTCCTTC |
|  | Intron 6_1 | Sense | CACCGATCCAGGGTACCGATTGGCA |
|  |  | Antisense | AAACTGCCAATCGGTACCCTGGATC |
|  | Intron 6_2 | Sense | CACCGTGCTGAATGAACAGTACAC |
|  |  | Antisense | AAACGTGTACTGTTCATTCAGCAC |

**Supplementary Table 2**

Primers for knock-in junction PCR in PITCh replacement

| **Locus** | **Junction** | **Direction** | **Sequence (5′ to 3′)** | **Amplicon size (bp)** |
| --- | --- | --- | --- | --- |
| *GUSB* | 5 prime | F | GCACCTCCCGCGCTTTTCTTAG | 464 |
|  |  | R | CATTCACCCACACGATGGCATAGGAAT |  |
|  | 3 prime | F | AGAGGAAGTCTGCTAACATGCGGTGAC | 946 |
|  |  | R | GCTGTGGAAGTCGCCCTGACTCG |  |
| *ARSB* | 5 prime | F | GCAAATTTAATTGCCGGGGAAGATAAC | 686 |
|  |  | R | CAGGAGTTTTTCATCCAGAGGAACACAG |  |
|  | 3 prime | F | AGAGGAAGTCTGCTAACATGCGGTGAC | 932 |
|  |  | R | TGGGATAACAAATGAGACAAGAGTCGTGAG |  |

Primers for knock-in junction PCR in REMOVER-PITCh

| **Locus** | **Junction** | **Direction** | **Sequence (5′ to 3′)** | **Amplicon size (bp)** |
| --- | --- | --- | --- | --- |
| *GUSB* | 5 prime | F | GCACCTCCCGCGCTTTTCTTAG | 640 |
|  |  | R | CTTGGAGGTGTCAGTCAGGTATTGGATG |  |
|  | 3 prime | F | GTTCTTTTTGTCAAGACCGACCTGTCC | 909 |
|  |  | R | GCCACTTTCATGCCAACTCTTTATTTCC |  |
| *ARSB* | 5 prime | F | GGCAGCCCAGTTCCTCATTCTATCAG | 465 |
|  |  | R | CAGGAGTTTTTCATCCAGAGGAACACAG |  |
|  | 3 prime-1 | F | GTTTCTGAGATACCCTCATCAGACC | 1,169 |
|  |  | R | TGGGATAACAAATGAGACAAGAGTCGTGAG |  |
|  | 3 prime-2 | F | GTTCTTTTTGTCAAGACCGACCTGTCC | 749 |
|  |  | R | TGGGATAACAAATGAGACAAGAGTCGTGAG |  |

Primers for long PCR

| **Locus** | **Junction** | **Direction** | **Sequence (5′ to 3′)** | **Amplicon size (bp)** |
| --- | --- | --- | --- | --- |
| *GUSB* | 5 prime long (out-in) | F | GCACCTCCCGCGCTTTTCTTAG | 2,328 |
|  |  | R | TCAGTGACAACGTCGAGCACAGC |  |
|  | 3 prime long (in-out) | F | GGCCGCTGTGGGAGTCAGG | 2,861 |
|  |  | R | GCCACTTTCATGCCAACTCTTTATTTCC |  |
| *ARSB* | 5 prime long (out-in) | F | GGCAGCCCAGTTCCTCATTCTATCAG | 1,994 |
|  |  | R | TCAGTGACAACGTCGAGCACAGC |  |
|  | 5 prime long (out-exon3) | F | GCAAATTTAATTGCCGGGGAAGATAAC | 914 |
|  |  | R | TCCTGTTGCAACTTCTTCGCCATC |  |
|  | 5 prime long (out-exon4) | F | GCAAATTTAATTGCCGGGGAAGATAAC | 1,145 |
|  |  | R | TGCAGTGACATTTCCTACTGCTTCATCC |  |
|  | 5 prime long (out-exon5) | F | GCAAATTTAATTGCCGGGGAAGATAAC | 1,421 |
|  |  | R | GAAGCCATCCAGAGGCTTTGTGC |  |
|  | 3 prime long (in-out) | F | CCGCTACCAGATCCGTACAGGTTTACAG | 2,241 |
|  |  | R | TGGGATAACAAATGAGACAAGAGTCGTGAG |  |
|  | 3 prime long (exon5-out) | F | CTTCGAGGAAGAAAATGGAGCCTGTG | 1,607 |
|  |  | R | TGGGATAACAAATGAGACAAGAGTCGTGAG |  |
|  | 3 prime long (exon6-out) | F | CAGAATTGAGCTGCTGCATAATATTGACC | 1,386 |
|  |  | R | TGGGATAACAAATGAGACAAGAGTCGTGAG |  |
|  | 3 prime long (exon7-out) | F | ATGACTCTTCTCTTCCAGAATATTCAGCCTTTAAC | 1,303 |
|  |  | R | TGGGATAACAAATGAGACAAGAGTCGTGAG |  |

Primers for out-out PCR in PITCh-replacement

| **Locus** | **Direction** | **Sequence (5′ to 3′)** | **Amplicon size (bp) (KI/Chromosomally deleted allele)** |
| --- | --- | --- | --- |
| *GUSB* | F | GCACCTCCCGCGCTTTTCTTAG | 2,983 / 231 |
|  | R | CTTGTTCTGCTGCTGTGGAAGTCG |  |
| *ARSB* | F | CAAGCGTCAGCTGAGTTTCCAAGAAG | 2,802 / 357 |
|  | R | TGGGATAACAAATGAGACAAGAGTCGTGAG |  |

Primers for out-out PCR in REMOVER-PITCh

| **Locus** | **Direction** | **Sequence (5′ to 3′)** | **Amplicon size (bp) (KI/Chromosomally deleted allele)** |
| --- | --- | --- | --- |
| *GUSB* | F | GCACCTCCCGCGCTTTTCTTAG | 3,115 / 366 |
|  | R | GCCACTTTCATGCCAACTCTTTATTTCC |  |
| *ARSB* | F | GGCAGCCCAGTTCCTCATTCTATCAG | 2,621 / 179 |
|  | R | TGGGATAACAAATGAGACAAGAGTCGTGAG |  |

Primers for non-knock-in allele

| **Locus** | **Region** | **Direction** | **Sequence (5′ to 3′)** | **Amplicon size (bp)** |
| --- | --- | --- | --- | --- |
| GUSB | 5'UTR - intron1 | F | GCACCTCCCGCGCTTTTCTTAG | 1,290 |
|  |  | R | GCCACTTTCATGCCAACTCTTTATTTCC |  |
|  | intron11 - 3'UTR | F | GCACCTCCCGCGCTTTTCTTAG | 431 |
|  |  | R | GCCACTTTCATGCCAACTCTTTATTTCC |  |
| ARSB | 5'UTR - intron1 | F | GGCAGCCCAGTTCCTCATTCTATCAG | 695 |
|  |  | R | AGCACCCGGCATTCCCATAAAC |  |
|  | intron7 - 3'UTR | F | AGAAGTCAAGTCTGAGAAGCATCTAGAGACAGC | 513 |
|  |  | R | TGGGATAACAAATGAGACAAGAGTCGTGAG |  |

**Supplementary Table 3**

Primers for the amplification of the off-target candidate sites

| **Gene** | **Locus** | **Direction** | **Sequence　(5′ to 3′)** | **Amplicon size (bp)** |
| --- | --- | --- | --- | --- |
| *GUSB* | #1 | F | GGTTTTGAAGACTTGGTAGGAATAAAAGATTAGCC | 408 |
|  |  | R | TGTCAGTGGTGTGCAATACAAATATAACATAAGTC |  |
|  | #2 | F | GAGAAGTGGGAGAATGAGAGGAAAAGAGAACATC | 442 |
|  |  | R | TGTCATTACCATCTTTGAGCCCTCCAG |  |
|  | #3 | F | CTCCATATATATACACACACATGCTCACCACACAC | 490 |
|  |  | R | ATTTCAATCCCATGGCAGCAAAATGTC |  |
|  | #4 | F | TCTACCTCTCTGTCCTTGTCTGGCTCTCC | 473 |
|  |  | R | CACATCCCCTGAGGTCTTCAACTCACC |  |
|  | #5 | F | CTAGTTTTAGCCAAGAACAGATCGACAGAAGC | 419 |
|  |  | R | AAAGGGAATTCAGGTACACAGACACACACAG |  |
|  | #6 | F | GGCAGCCCTAGCTTCCAACTGC | 394 |
|  |  | R | GATGTATTGGGTCTGGTTTGGTGGAAAG |  |
|  | #7 | F | CCTGATTCAACTTTACATTCCTTCCACCATTATC | 411 |
|  |  | R | TTATTTGCCAGAGGAAATAGCTTCTCTACCCTCTAC |  |

| **Gene** | **Locus** | **Direction** | **Sequence　(5′ to 3′)** | **Amplicon size (bp)** |
| --- | --- | --- | --- | --- |
| *ARSB* | #1 | F | GATAGCAAGCTTAAGTCACAAATACAAATTATGAGC | 440 |
|  |  | R | CTTTCCCTGAGGGCTGAGGTATCTG |  |
|  | #2 | F | CCGCAGACTCGAGATAAAGGAGAGGAG | 450 |
|  |  | R | CACTTCTCCTTTGCCTTCTAGTGCTTTCTTC |  |
|  | #3 | F | CCCGAGTGGAGAGTGGAGACGAATC | 308 |
|  |  | R | TCGGCGCTGCTGCTACTGTTGTC |  |
|  | #4 | F | GTGGCAGAGCCTCAGAATGGAAATC | 454 |
|  |  | R | GCAGTCTACGTTGTTCACCAGCAGAG |  |
|  | #5 | F | TGGTTCTGAAGAGTGGGGAGTAAGGAG | 478 |
|  |  | R | GACTCCCCTATCAGCTTTCACCCTTTC |  |
|  | #6 | F | TCCCACCCCAGACAACATTTTTAAGC | 396 |
|  |  | R | GAGGTACAGACCTGTTGCTTGCCTGAAC |  |
|  | #7 | F | ACCATGAGGTACCCCAACTATCTTGGATCTC | 353 |
|  |  | R | GTTTCAGAATCCCTGGGTACTCCTCATTTC |  |

Primers for sequencing analysis of off-target candidate sites

| **Gene** | **Locus** | **Sequence (5′ to 3′)** |
| --- | --- | --- |
| *GUSB* | #1 | CCAAACATTAACTAAAGTGTACAGACCTTG |
|  | #2 | TGTGAGCTCTGTGAGGGTAGG |
|  | #3 | GTTTAGGAGCAATGTGGTTGC |
|  | #4 | GCTTGTCACACGTCCTCAAAC |
|  | #5 | CCATGCAGAACCTAAGACGATG |
|  | #6 | CTCAAATCTGCGTCTTTGTTCTG |
|  | #7 | GATTAATCCACGCTAGCATTCC |

| **Gene** | **Locus** | **Sequence (5′ to 3′)** |
| --- | --- | --- |
| *ARSB* | #1 | CTTGCTGTCCATAGTCTCAGGTTAC |
|  | #2 | ACCTCTTTGATGCACTCTCACG |
|  | #3 | TTCCCATGCAAACCCACTTAG |
|  | #4 | GCTGGTGGAAAGAGCAGAGTAAG |
|  | #5 | CTGCTTTCTTGGTTCTGAAGAGTG |
|  | #6 | TCCCAACTAGGATCTCCAGCTAC |
|  | #7 | GCTGTTGAGTTGAGGATTTCTGAG |

**Supplementary Sequences**

***Donor constructs***

**PITCh-donor for PITCh-replacement at the *GUSB* locus**

**PITCh sgRNA-Left microhomology-*hGUSB* CDS-T2A-NeoR-Right micorohomology-PITCh sgRNA**

**(Silent mutation)**

**TCGCGCGTTTCGGTGATGACGGTGAAAACCTCTGACACATGCAGCTCCCGGAGACGGTCACAGCTTGTCTGTAAGCGGATGCCGGGAGCAGACAAGCCCGTCAGGGCGCGTCAGCGGGTGTTGGCGGGTGTCGGGGCTGGCTTAACTATGCGGCATCAGAGCAGATTGTACTGAGAGTGCACCATATGCGGTGTGAAATACCGCACAGATGCGTAAGGAGAAAATATTACTACAGGCGCCATTCGCCATTCAGGCTGCGCAACTGTTGGGAAGGGCGATCGGTGCGGGCCTCTTCGCTATTACGCCAGCTGGCGAAAGGGGGATGTGCTGCAAGGCGATTAAGTTGGGTAACGCCAGGGTTTTCCCAGTCACGACGTTGTAAAACGACGGCCAGTGAGCTAGTGTAATACGACTCACTATAGGGCGCGGCCGCAGAATTCGAGCTCGGTACCCGGGATCTCGAGGCCAGATCGTGCTTCGATATCGATCGTTTGGcggcgctcgggccgttgttgtggggctgcgcgctggggctAcagggcgggatgctgtacccccaggagagcccgtcgcgggagtgcaaggagctggacggcctctggagcttccgcgccgacttctctgacaaccgacgccggggcttcgaggagcagtggtaccggcggccgctgtgggagtcaggccccaccgtggacatgccagttccctccagcttcaatgacatcagccaggactggcgtctgcggcattttgtcggctgggtgtggtacgaacgggaggtgatcctgccggagcgatggacccaggacctgcgcacaagagtggtgctgaggattggcagtgcccattcctatgccatcgtgtgggtgaatggggtcgacacgctagagcatgaggggggctacctccccttcgaggccgacatcagcaacctggtccaggtggggcccctgccctcccggctccgaatcactatcgccatcaacaacacactcacccccaccaccctgccaccagggaccatccaatacctgactgacacctccaagtatcccaagggttactttgtccagaacacatattttgactttttcaactacgctggactgcagcggtctgtacttctgtacacgacacccaccacctacatcgatgacatcaccgtcaccaccagcgtggagcaagacagtgggctggtgaattaccagatctctgtcaagggcagtaacctgttcaagttggaagtgcgtcttttggatgcagaaaacaaagtcgtggcgaatgggactgggacccagggccaacttaaggtgccaggtgtcagcctctggtggccgtacctgatgcacgaacgccctgcctatctgtattcattggaggtgcagctgactgcacagacgtcactggggcctgtgtctgacttctacacactccctgtggggatccgcactgtggctgtcaccaagagccagttcctcatcaatgggaaacctttctatttccacggtgtcaacaagcatgaggatgcggacatccgagggaagggcttcgactggccgctgctggtgaaggacttcaacctgcttcgctggcttggtgccaacgctttccgtaccagccactacccctatgcagaggaagtgatgcagatgtgtgaccgctatgggattgtggtcatcgatgagtgtcccggcgtgggcctggcgctgccgcagttcttcaacaacgtttctctgcatcaccacatgcaggtgatggaagaagtggtgcgtagggacaagaaccaccccgcggtcgtgatgtggtctgtggccaacgagcctgcgtcccacctagaatctgctggctactacttgaagatggtgatcgctcacaccaaatccttggacccctcccggcctgtgacctttgtgagcaactctaactatgcagcagacaagggggctccgtatgtggatgtgatctgtttgaacagctactactcttggtatcacgactacgggcacctggagttgattcagctgcagctggccacccagtttgagaactggtataagaagtatcagaagcccattattcagagcgagtatggagcagaaacgattgcagggtttcaccaggatccacctctgatgttcactgaagagtaccagaaaagtctgctagagcagtaccatctgggtctggatcaaaaacgcagaaaatacgtggttggagagctcatttggaattttgccgatttcatgactgaacagtcaccgacgagagtgctggggaataaaaaggggatcttcactcggcagagacaaccaaaaagtgcagcgttccttttgcgagagagatactggaagattgccaatgaaaccaggtatccccaTAGCgtGgcTaagtcacaatgtttggaaaacagccCgtttactGGAAGCGGAGAGGGCAGAGGAAGTCTGCTAACATGCGGTGACGTCGAGGAGAATCCTGGACCTATGATTGAACAAGATGGATTGCACGCAGGTTCTCCGGCCGCTTGGGTGGAGAGGCTATTCGGCTATGACTGGGCACAACAGACAATCGGCTGCTCTGATGCCGCCGTGTTCCGGCTGTCAGCGCAGGGGCGCCCGGTTCTTTTTGTCAAGACCGACCTGTCCGGTGCCCTGAATGAACTGCAGGACGAGGCAGCGCGGCTATCGTGGCTGGCCACGACGGGCGTTCCTTGCGCAGCTGTGCTCGACGTTGTCACTGAAGCGGGAAGGGACTGGCTGCTATTGGGCGAAGTGCCGGGGCAGGATCTCCTGTCATCTCACCTTGCTCCTGCCGAGAAAGTATCCATCATGGCTGATGCAATGCGGCGGCTGCATACGCTTGATCCGGCTACCTGCCCATTCGACCACCAAGCGAAACATCGCATCGAGCGAGCACGTACTCGGATGGAAGCCGGTCTTGTCGATCAGGATGATCTGGACGAAGAGCATCAGGGGCTCGCGCCAGCCGAACTGTTCGCCAGGCTCAAGGCGCGCATGCCCGACGGCGAGGATCTCGTCGTGACCCATGGCGATGCCTGCTTGCCGAATATCATGGTGGAAAATGGCCGCTTTTCTGGATTCATCGACTGTGGCCGGCTGGGTGTGGCGGACCGCTATCAGGACATAGCGTTGGCTACCCGTGATATTGCTGAAGAGCTTGGCGGCGAATGGGCTGACCGCTTCCTCGTGCTTTACGGTATCGCCGCTCCCGATTCGCAGCGCATCGCCTTCTATCGCCTTCTTGACGAGTTCTTCTGAtcagtagccaagtcacaatgtttggaaaacagccCgtttaCCAAACGATCGATATCGAAGCACATTGTGGATCCGCTCTAGAGTCGACCTGCAGGCATGCAAGCTTGCGGCCGCGTATTCTATAGTGTCACCTAAATAGCATGGCGTAATCATGGTCATAGCTGTTTCCTGTGTGAAATTGTTATCCGCTCACAATTCCACACAACATACGAGCCGGAAGCATAAAGTGTAAAGCCTGGGGTGCCTAATGAGTGAGCTAACTCACATTAATTGCGTTGCGCTCACTGCCCGCTTTCCAGTCGGGAAACCTGTCGTGCCAGCTGCATTAATGAATCGGCCAACGCGCGGGGAGAGGCGGTTTGCGTATTGGGCGCTCTTCCGCTTCCTCGCTCACTGACTCGCTGCGCTCGGTCGTTCGGCTGCGGCGAGCGGTATCAGCTCACTCAAAGGCGGTAATACGGTTATCCACAGAATCAGGGGATAACGCAGGAAAGAACATGTGAGCAAAAGGCCAGCAAAAGGCCAGGAACCGTAAAAAGGCCGCGTTGCTGGCGTTTTTCCATAGGCTCCGCCCCCCTGACGAGCATCACAAAAATCGACGCTCAAGTCAGAGGTGGCGAAACCCGACAGGACTATAAAGATACCAGGCGTTTCCCCCTGGAAGCTCCCTCGTGCGCTCTCCTGTTCCGACCCTGCCGCTTACCGGATACCTGTCCGCCTTTCTCCCTTCGGGAAGCGTGGCGCTTTCTCATAGCTCACGCTGTAGGTATCTCAGTTCGGTGTAGGTCGTTCGCTCCAAGCTGGGCTGTGTGCACGAACCCCCCGTTCAGCCCGACCGCTGCGCCTTATCCGGTAACTATCGTCTTGAGTCCAACCCGGTAAGACACGACTTATCGCCACTGGCAGCAGCCACTGGTAACAGGATTAGCAGAGCGAGGTATGTAGGCGGTGCTACAGAGTTCTTGAAGTGGTGGCCTAACTACGGCTACACTAGAAGAACAGTATTTGGTATCTGCGCTCTGCTGAAGCCAGTTACCTTCGGAAAAAGAGTTGGTAGCTCTTGATCCGGCAAACAAACCACCGCTGGTAGCGGTGGTTTTTTTGTTTGCAAGCAGCAGATTACGCGCAGAAAAAAAGGATCTCAAGAAGATCCTTTGATCTTTTCTACGGGGTCTGACGCTCAGTGGAACGAAAACTCACGTTAAGGGATTTTGGTCATGAGATTATCAAAAAGGATCTTCACCTAGATCCTTTTAAATTAAAAATGAAGTTTTAAATCAATCTAAAGTATATATGTTTAAACTTGGTCTGACAGTTACCAATGCTTAATCAGTGAGGCACCTATCTCAGCGATCTGTCTATTTCGTTCATCCATAGTTGCCTGACTCCCCGTCGTGTAGATAACTACGATACGGGAGGGCTTACCATCTGGCCCCAGTGCTGCAATGATACCGCGAGACCCACGCTCACCGGCTCCAGATTTATCAGCAATAAACCAGCCAGCCGGAAGGGCCGAGCGCAGAAGTGGTCCTGCAACTTTATCCGCCTCCATCCAGTCTATTAATTGTTGCCGGGAAGCTAGAGTAAGTAGTTCGCCAGTTAATAGTTTGCGCAACGTTGTTGCCATTGCTACAGGCATCGTGGTGTCACGCTCGTCGTTTGGTATGGCTTCATTCAGCTCCGGTTCCCAACGATCAAGGCGAGTTACATGATCCCCCATGTTGTGCAAAAAAGCGGTTAGCTCCTTCGGTCCTCCGATCGTTGTCAGAAGTAAGTTGGCCGCAGTGTTATCACTCATGGTTATGGCAGCACTGCATAATTCTCTTACTGTCATGCCATCCGTAAGATGCTTTTCTGTGACTGGTGAGTACTCAACCAAGTCATTCTGAGAATAGTGTATGCGGCGACCGAGTTGCTCTTGCCCGGCGTCAATACGGGATAATACCGCGCCACATAGCAGAACTTTAAAAGTGCTCATCATTGGAAAACGTTCTTCGGGGCGAAAACTCTCAAGGATCTTACCGCTGTTGAGATCCAGTTCGATGTAACCCACTCGTGCACCCAACTGATCTTCAGCATCTTTTACTTTCACCAGCGTTTCTGGGTGAGCAAAAACAGGAAGGCAAAATGCCGCAAAAAAGGGAATAAGGGCGACACGGAAATGTTGAATACTCATACTCTTCCTTTTTCAATATTATTATAAGCATTTATCAGGGTTATTGTCTCATGAGCGGATACATATTTGAATGTATTTAGAAAAATAAACAAATAGGGGTTCCGCGCACATTTCCCCGAAAAGTGCCACCTGACGTCTAAGAAACCATTATTATCATGACATTAACCTATAAAAATAGGCGTATCACGAGGCCCTTTCGTC**

**PITCh-donor for PITCh-replacement at the *ARSB* locus**

**PITCh sgRNA-Left microhomology-*hARSB* CDS-T2A-NeoR-Right micorohomology-PITCh sgRNA**

**(Silent mutation)**

**TCGCGCGTTTCGGTGATGACGGTGAAAACCTCTGACACATGCAGCTCCCGGAGACGGTCACAGCTTGTCTGTAAGCGGATGCCGGGAGCAGACAAGCCCGTCAGGGCGCGTCAGCGGGTGTTGGCGGGTGTCGGGGCTGGCTTAACTATGCGGCATCAGAGCAGATTGTACTGAGAGTGCACCATATGCGGTGTGAAATACCGCACAGATGCGTAAGGAGAAAATATTACTACAGGCGCCATTCGCCATTCAGGCTGCGCAACTGTTGGGAAGGGCGATCGGTGCGGGCCTCTTCGCTATTACGCCAGCTGGCGAAAGGGGGATGTGCTGCAAGGCGATTAAGTTGGGTAACGCCAGGGTTTTCCCAGTCACGACGTTGTAAAACGACGGCCAGTGAGCTAGTGTAATACGACTCACTATAGGGCGCGGCCGCAGAATTCGAGCTCGGTACCCGGGATCTCGAGGCCAGATCGTGCTTCGATATCGATCGTTTGGcaggcgctgggaccgcgggcggacaaggatgggtccgcgcggGgcCgcCTCTCTCccccgaggccccggacctcggcggctgctcctccccgtcgtcctcccgctgctgctgctgctgttgttggcgccgccgggctcgggcgccggggccagccggccgccccacctggtcttcttgctggcagacgacctaggctggaacgacgtcggcttccacggctcccgcatccgcacgccgcacctggacgcgctggcggccggcggggtgctcctggacaactactacacgcagccgctgtgcacgccgtcgcggagccagctgctcactggccgctaccagatccgtacaggtttacagcaccaaataatctggccctgtcagcccagctgtgttcctctggatgaaaaactcctgccccagctcctaaaagaagcaggttatactacccatatggtcggaaaatggcacctgggaatgtaccggaaagaatgccttccaacccgccgaggatttgatacctactttggatatctcctgggtagtgaagattattattcccatgaacgctgtacattaattgacgctctgaatgtcacacgatgtgctcttgattttcgagatggcgaagaagttgcaacaggatataaaaatatgtattcaacaaacatattcaccaaaagggctatagccctcataactaaccatccaccagagaagcctctgtttctctaccttgctctccagtctgtgcatgagccccttcaggtccctgaggaatacttgaagccatatgactttatccaagacaagaacaggcatcactatgcaggaatggtgtcccttatggatgaagcagtaggaaatgtcactgcagctttaaaaagcagtgggctctggaacaacacggtgttcatcttttctacagataacggagggcagactttggcagggggtaataactggccccttcgaggaagaaaatggagcctgtgggaaggaggcgtccgaggggtgggctttgtggcaagccccttgctgaagcagaagggcgtgaagaaccgggagctcatccacatctctgactggctgccaacactcgtgaagctggccaggggacacaccaatggcacaaagcctctggatggcttcgacgtgtggaaaaccatcagtgaaggaagcccatcccccagaattgagctgctgcataatattgacccAaacttcgtggactcttcaccgtgtcccaggaacagcatggctccagcaaaggatgactcttctcttccagaatattcagcctttaacacatctgtccatgctgcaattagacatggaaattggaaactcctcacgggctacccaggctgtggttactggttccctccaccAtctcaatacaatgtttctgagataccctcatcagacccaccaaccaagaccctctggctctttgatattgatcgggaccctgaagaaagacatgacctgtccagagaatatcctcacatcgtcacaaagctcctgtcccgcctacagttctaccataaacactcagtccccgtgtacttccctgcacaggacccccgctgtgatcccaaggccactggggtgtggggcccttggatgGGAAGCGGAGAGGGCAGAGGAAGTCTGCTAACATGCGGTGACGTCGAGGAGAATCCTGGACCTATGATTGAACAAGATGGATTGCACGCAGGTTCTCCGGCCGCTTGGGTGGAGAGGCTATTCGGCTATGACTGGGCACAACAGACAATCGGCTGCTCTGATGCCGCCGTGTTCCGGCTGTCAGCGCAGGGGCGCCCGGTTCTTTTTGTCAAGACCGACCTGTCCGGTGCCCTGAATGAACTGCAGGACGAGGCAGCGCGGCTATCGTGGCTGGCCACGACGGGCGTTCCTTGCGCAGCTGTGCTCGACGTTGTCACTGAAGCGGGAAGGGACTGGCTGCTATTGGGCGAAGTGCCGGGGCAGGATCTCCTGTCATCTCACCTTGCTCCTGCCGAGAAAGTATCCATCATGGCTGATGCAATGCGGCGGCTGCATACGCTTGATCCGGCTACCTGCCCATTCGACCACCAAGCGAAACATCGCATCGAGCGAGCACGTACTCGGATGGAAGCCGGTCTTGTCGATCAGGATGATCTGGACGAAGAGCATCAGGGGCTCGCGCCAGCCGAACTGTTCGCCAGGCTCAAGGCGCGCATGCCCGACGGCGAGGATCTCGTCGTGACCCATGGCGATGCCTGCTTGCCGAATATCATGGTGGAAAATGGCCGCTTTTCTGGATTCATCGACTGTGGCCGGCTGGGTGTGGCGGACCGCTATCAGGACATAGCGTTGGCTACCCGTGATATTGCTGAAGAGCTTGGCGGCGAATGGGCTGACCGCTTCCTCGTGCTTTACGGTATCGCCGCTCCCGATTCGCAGCGCATCGCCTTCTATCGCCTTCTTGACGAGTTCTTCTGAtgtaggatttcagggaggctagaaaacctttcaattggaaCCAAACGATCGATATCGAAGCACATTGTGGATCCGCTCTAGAGTCGACCTGCAGGCATGCAAGCTTGCGGCCGCGTATTCTATAGTGTCACCTAAATAGCATGGCGTAATCATGGTCATAGCTGTTTCCTGTGTGAAATTGTTATCCGCTCACAATTCCACACAACATACGAGCCGGAAGCATAAAGTGTAAAGCCTGGGGTGCCTAATGAGTGAGCTAACTCACATTAATTGCGTTGCGCTCACTGCCCGCTTTCCAGTCGGGAAACCTGTCGTGCCAGCTGCATTAATGAATCGGCCAACGCGCGGGGAGAGGCGGTTTGCGTATTGGGCGCTCTTCCGCTTCCTCGCTCACTGACTCGCTGCGCTCGGTCGTTCGGCTGCGGCGAGCGGTATCAGCTCACTCAAAGGCGGTAATACGGTTATCCACAGAATCAGGGGATAACGCAGGAAAGAACATGTGAGCAAAAGGCCAGCAAAAGGCCAGGAACCGTAAAAAGGCCGCGTTGCTGGCGTTTTTCCATAGGCTCCGCCCCCCTGACGAGCATCACAAAAATCGACGCTCAAGTCAGAGGTGGCGAAACCCGACAGGACTATAAAGATACCAGGCGTTTCCCCCTGGAAGCTCCCTCGTGCGCTCTCCTGTTCCGACCCTGCCGCTTACCGGATACCTGTCCGCCTTTCTCCCTTCGGGAAGCGTGGCGCTTTCTCATAGCTCACGCTGTAGGTATCTCAGTTCGGTGTAGGTCGTTCGCTCCAAGCTGGGCTGTGTGCACGAACCCCCCGTTCAGCCCGACCGCTGCGCCTTATCCGGTAACTATCGTCTTGAGTCCAACCCGGTAAGACACGACTTATCGCCACTGGCAGCAGCCACTGGTAACAGGATTAGCAGAGCGAGGTATGTAGGCGGTGCTACAGAGTTCTTGAAGTGGTGGCCTAACTACGGCTACACTAGAAGAACAGTATTTGGTATCTGCGCTCTGCTGAAGCCAGTTACCTTCGGAAAAAGAGTTGGTAGCTCTTGATCCGGCAAACAAACCACCGCTGGTAGCGGTGGTTTTTTTGTTTGCAAGCAGCAGATTACGCGCAGAAAAAAAGGATCTCAAGAAGATCCTTTGATCTTTTCTACGGGGTCTGACGCTCAGTGGAACGAAAACTCACGTTAAGGGATTTTGGTCATGAGATTATCAAAAAGGATCTTCACCTAGATCCTTTTAAATTAAAAATGAAGTTTTAAATCAATCTAAAGTATATATGTTTAAACTTGGTCTGACAGTTACCAATGCTTAATCAGTGAGGCACCTATCTCAGCGATCTGTCTATTTCGTTCATCCATAGTTGCCTGACTCCCCGTCGTGTAGATAACTACGATACGGGAGGGCTTACCATCTGGCCCCAGTGCTGCAATGATACCGCGAGACCCACGCTCACCGGCTCCAGATTTATCAGCAATAAACCAGCCAGCCGGAAGGGCCGAGCGCAGAAGTGGTCCTGCAACTTTATCCGCCTCCATCCAGTCTATTAATTGTTGCCGGGAAGCTAGAGTAAGTAGTTCGCCAGTTAATAGTTTGCGCAACGTTGTTGCCATTGCTACAGGCATCGTGGTGTCACGCTCGTCGTTTGGTATGGCTTCATTCAGCTCCGGTTCCCAACGATCAAGGCGAGTTACATGATCCCCCATGTTGTGCAAAAAAGCGGTTAGCTCCTTCGGTCCTCCGATCGTTGTCAGAAGTAAGTTGGCCGCAGTGTTATCACTCATGGTTATGGCAGCACTGCATAATTCTCTTACTGTCATGCCATCCGTAAGATGCTTTTCTGTGACTGGTGAGTACTCAACCAAGTCATTCTGAGAATAGTGTATGCGGCGACCGAGTTGCTCTTGCCCGGCGTCAATACGGGATAATACCGCGCCACATAGCAGAACTTTAAAAGTGCTCATCATTGGAAAACGTTCTTCGGGGCGAAAACTCTCAAGGATCTTACCGCTGTTGAGATCCAGTTCGATGTAACCCACTCGTGCACCCAACTGATCTTCAGCATCTTTTACTTTCACCAGCGTTTCTGGGTGAGCAAAAACAGGAAGGCAAAATGCCGCAAAAAAGGGAATAAGGGCGACACGGAAATGTTGAATACTCATACTCTTCCTTTTTCAATATTATTATAAGCATTTATCAGGGTTATTGTCTCATGAGCGGATACATATTTGAATGTATTTAGAAAAATAAACAAATAGGGGTTCCGCGCACATTTCCCCGAAAAGTGCCACCTGACGTCTAAGAAACCATTATTATCATGACATTAACCTATAAAAATAGGCGTATCACGAGGCCCTTTCGTC**

PITCh-donor for REMOVER-PITCh at the *GUSB* locus

**PITCh sgRNA-Left microhomology-*hGUSB* CDS-T2A-NeoR-Rightmicorohomology-PITCh sgRNA**

**(Silent mutation)**

**TCGCGCGTTTCGGTGATGACGGTGAAAACCTCTGACACATGCAGCTCCCGGAGACGGTCACAGCTTGTCTGTAAGCGGATGCCGGGAGCAGACAAGCCCGTCAGGGCGCGTCAGCGGGTGTTGGCGGGTGTCGGGGCTGGCTTAACTATGCGGCATCAGAGCAGATTGTACTGAGAGTGCACCATATGCGGTGTGAAATACCGCACAGATGCGTAAGGAGAAAATATTACTACAGGCGCCATTCGCCATTCAGGCTGCGCAACTGTTGGGAAGGGCGATCGGTGCGGGCCTCTTCGCTATTACGCCAGCTGGCGAAAGGGGGATGTGCTGCAAGGCGATTAAGTTGGGTAACGCCAGGGTTTTCCCAGTCACGACGTTGTAAAACGACGGCCAGTGAGCTAGTGTAATACGACTCACTATAGGGCGCGGCCGCAGAATTCGAGCTCGGTACCCGGGATCTCGAGGCCAGATCGTGCTTCGATATCGATCGTTTGGttgcctgggcggcgctcgggccgttgttgtggggctgcgcCTtggggctgcagggcgggatgctgtacccccaggagagcccgtcgcgggagtgcaaggagctggacggcctctggagcttccgcgccgacttctctgacaaccgacgccggggcttcgaggagcagtggtaccggcggccgctgtgggagtcaggccccaccgtggacatgccagttccctccagcttcaatgacatcagccaggactggcgtctgcggcattttgtcggctgggtgtggtacgaacgggaggtgatcctgccggagcgatggacccaggacctgcgcacaagagtggtgctgaggattggcagtgcccattcctatgccatcgtgtgggtgaatggggtcgacacgctagagcatgaggggggctacctccccttcgaggccgacatcagcaacctggtccaggtggggcccctgccctcccggctccgaatcactatcgccatcaacaacacactcacccccaccaccctgccaccagggaccatccaatacctgactgacacctccaagtatcccaagggttactttgtccagaacacatattttgactttttcaactacgctggactgcagcggtctgtacttctgtacacgacacccaccacctacatcgatgacatcaccgtcaccaccagcgtggagcaagacagtgggctggtgaattaccagatctctgtcaagggcagtaacctgttcaagttggaagtgcgtcttttggatgcagaaaacaaagtcgtggcgaatgggactgggacccagggccaacttaaggtgccaggtgtcagcctctggtggccgtacctgatgcacgaacgccctgcctatctgtattcattggaggtgcagctgactgcacagacgtcactggggcctgtgtctgacttctacacactccctgtggggatccgcactgtggctgtcaccaagagccagttcctcatcaatgggaaacctttctatttccacggtgtcaacaagcatgaggatgcggacatccgagggaagggcttcgactggccgctgctggtgaaggacttcaacctgcttcgctggcttggtgccaacgctttccgtaccagccactacccctatgcagaggaagtgatgcagatgtgtgaccgctatgggattgtggtcatcgatgagtgtcccggcgtgggcctggcgctgccgcagttcttcaacaacgtttctctgcatcaccacatgcaggtgatggaagaagtggtgcgtagggacaagaaccaccccgcggtcgtgatgtggtctgtggccaacgagcctgcgtcccacctagaatctgctggctactacttgaagatggtgatcgctcacaccaaatccttggacccctcccggcctgtgacctttgtgagcaactctaactatgcagcagacaagggggctccgtatgtggatgtgatctgtttgaacagctactactcttggtatcacgactacgggcacctggagttgattcagctgcagctggccacccagtttgagaactggtataagaagtatcagaagcccattattcagagcgagtatggagcagaaacgattgcagggtttcaccaggatccacctctgatgttcactgaagagtaccagaaaagtctgctagagcagtaccatctgggtctggatcaaaaacgcagaaaatacgtggttggagagctcatttggaattttgccgatttcatgactgaacagtcaccgacgagagtgctggggaataaaaaggggatcttcactcggcagagacaaccaaaaagtgcagcgttccttttgcgagagagatactggaagattgccaatgaaaccaggtatccccaTAGCgtGgcTaagtcacaatgtttggaaaacagccCgtttactGGAAGCGGAGAGGGCAGAGGAAGTCTGCTAACATGCGGTGACGTCGAGGAGAATCCTGGACCTATTGAACAAGATGGATTGCACGCAGGTTCTCCGGCCGCTTGGGTGGAGAGGCTATTCGGCTATGACTGGGCACAACAGACAATCGGCTGCTCTGATGCCGCCGTGTTCCGGCTGTCAGCGCAGGGGCGCCCGGTTCTTTTTGTCAAGACCGACCTGTCCGGTGCCCTGAATGAACTGCAGGACGAGGCAGCGCGGCTATCGTGGCTGGCCACGACGGGCGTTCCTTGCGCAGCTGTGCTCGACGTTGTCACTGAAGCGGGAAGGGACTGGCTGCTATTGGGCGAAGTGCCGGGGCAGGATCTCCTGTCATCTCACCTTGCTCCTGCCGAGAAAGTATCCATCATGGCTGATGCAATGCGGCGGCTGCATACGCTTGATCCGGCTACCTGCCCATTCGACCACCAAGCGAAACATCGCATCGAGCGAGCACGTACTCGGATGGAAGCCGGTCTTGTCGATCAGGATGATCTGGACGAAGAGCATCAGGGGCTCGCGCCAGCCGAACTGTTCGCCAGGCTCAAGGCGCGCATGCCCGACGGCGAGGATCTCGTCGTGACCCATGGCGATGCCTGCTTGCCGAATATCATGGTGGAAAATGGCCGCTTTTCTGGATTCATCGACTGTGGCCGGCTGGGTGTGGCGGACCGCTATCAGGACATAGCGTTGGCTACCCGTGATATTGCTGAAGAGCTTGGCGGCGAATGGGCTGACCGCTTCCTCGTGCTTTACGGTATCGCCGCTCCCGATTCGCAGCGCATCGCCTTCTATCGCCTTCTTGACGAGTTCTTCTGAtcagtagccaagtcacaatgtttggaaaacagccCgtttaCCAAACGATCGATATCGAAGCACATTGTGGATCCGCTCTAGAGTCGACCTGCAGGCATGCAAGCTTGCGGCCGCGTATTCTATAGTGTCACCTAAATAGCATGGCGTAATCATGGTCATAGCTGTTTCCTGTGTGAAATTGTTATCCGCTCACAATTCCACACAACATACGAGCCGGAAGCATAAAGTGTAAAGCCTGGGGTGCCTAATGAGTGAGCTAACTCACATTAATTGCGTTGCGCTCACTGCCCGCTTTCCAGTCGGGAAACCTGTCGTGCCAGCTGCATTAATGAATCGGCCAACGCGCGGGGAGAGGCGGTTTGCGTATTGGGCGCTCTTCCGCTTCCTCGCTCACTGACTCGCTGCGCTCGGTCGTTCGGCTGCGGCGAGCGGTATCAGCTCACTCAAAGGCGGTAATACGGTTATCCACAGAATCAGGGGATAACGCAGGAAAGAACATGTGAGCAAAAGGCCAGCAAAAGGCCAGGAACCGTAAAAAGGCCGCGTTGCTGGCGTTTTTCCATAGGCTCCGCCCCCCTGACGAGCATCACAAAAATCGACGCTCAAGTCAGAGGTGGCGAAACCCGACAGGACTATAAAGATACCAGGCGTTTCCCCCTGGAAGCTCCCTCGTGCGCTCTCCTGTTCCGACCCTGCCGCTTACCGGATACCTGTCCGCCTTTCTCCCTTCGGGAAGCGTGGCGCTTTCTCATAGCTCACGCTGTAGGTATCTCAGTTCGGTGTAGGTCGTTCGCTCCAAGCTGGGCTGTGTGCACGAACCCCCCGTTCAGCCCGACCGCTGCGCCTTATCCGGTAACTATCGTCTTGAGTCCAACCCGGTAAGACACGACTTATCGCCACTGGCAGCAGCCACTGGTAACAGGATTAGCAGAGCGAGGTATGTAGGCGGTGCTACAGAGTTCTTGAAGTGGTGGCCTAACTACGGCTACACTAGAAGAACAGTATTTGGTATCTGCGCTCTGCTGAAGCCAGTTACCTTCGGAAAAAGAGTTGGTAGCTCTTGATCCGGCAAACAAACCACCGCTGGTAGCGGTGGTTTTTTTGTTTGCAAGCAGCAGATTACGCGCAGAAAAAAAGGATCTCAAGAAGATCCTTTGATCTTTTCTACGGGGTCTGACGCTCAGTGGAACGAAAACTCACGTTAAGGGATTTTGGTCATGAGATTATCAAAAAGGATCTTCACCTAGATCCTTTTAAATTAAAAATGAAGTTTTAAATCAATCTAAAGTATATATGTTTAAACTTGGTCTGACAGTTACCAATGCTTAATCAGTGAGGCACCTATCTCAGCGATCTGTCTATTTCGTTCATCCATAGTTGCCTGACTCCCCGTCGTGTAGATAACTACGATACGGGAGGGCTTACCATCTGGCCCCAGTGCTGCAATGATACCGCGAGACCCACGCTCACCGGCTCCAGATTTATCAGCAATAAACCAGCCAGCCGGAAGGGCCGAGCGCAGAAGTGGTCCTGCAACTTTATCCGCCTCCATCCAGTCTATTAATTGTTGCCGGGAAGCTAGAGTAAGTAGTTCGCCAGTTAATAGTTTGCGCAACGTTGTTGCCATTGCTACAGGCATCGTGGTGTCACGCTCGTCGTTTGGTATGGCTTCATTCAGCTCCGGTTCCCAACGATCAAGGCGAGTTACATGATCCCCCATGTTGTGCAAAAAAGCGGTTAGCTCCTTCGGTCCTCCGATCGTTGTCAGAAGTAAGTTGGCCGCAGTGTTATCACTCATGGTTATGGCAGCACTGCATAATTCTCTTACTGTCATGCCATCCGTAAGATGCTTTTCTGTGACTGGTGAGTACTCAACCAAGTCATTCTGAGAATAGTGTATGCGGCGACCGAGTTGCTCTTGCCCGGCGTCAATACGGGATAATACCGCGCCACATAGCAGAACTTTAAAAGTGCTCATCATTGGAAAACGTTCTTCGGGGCGAAAACTCTCAAGGATCTTACCGCTGTTGAGATCCAGTTCGATGTAACCCACTCGTGCACCCAACTGATCTTCAGCATCTTTTACTTTCACCAGCGTTTCTGGGTGAGCAAAAACAGGAAGGCAAAATGCCGCAAAAAAGGGAATAAGGGCGACACGGAAATGTTGAATACTCATACTCTTCCTTTTTCAATATTATTATAAGCATTTATCAGGGTTATTGTCTCATGAGCGGATACATATTTGAATGTATTTAGAAAAATAAACAAATAGGGGTTCCGCGCACATTTCCCCGAAAAGTGCCACCTGACGTCTAAGAAACCATTATTATCATGACATTAACCTATAAAAATAGGCGTATCACGAGGCCCTTTCGTC**

PITCh-donor for REMOVER-PITCh at the *ARSB* locus

**PITCh sgRNA-Left microhomology-*hARSB* CDS-T2A-NeoR-Right micorohomology-PITCh sgRNA**

**(Silent mutation)**

**TCGCGCGTTTCGGTGATGACGGTGAAAACCTCTGACACATGCAGCTCCCGGAGACGGTCACAGCTTGTCTGTAAGCGGATGCCGGGAGCAGACAAGCCCGTCAGGGCGCGTCAGCGGGTGTTGGCGGGTGTCGGGGCTGGCTTAACTATGCGGCATCAGAGCAGATTGTACTGAGAGTGCACCATATGCGGTGTGAAATACCGCACAGATGCGTAAGGAGAAAATATTACTACAGGCGCCATTCGCCATTCAGGCTGCGCAACTGTTGGGAAGGGCGATCGGTGCGGGCCTCTTCGCTATTACGCCAGCTGGCGAAAGGGGGATGTGCTGCAAGGCGATTAAGTTGGGTAACGCCAGGGTTTTCCCAGTCACGACGTTGTAAAACGACGGCCAGTGAGCTAGTGTAATACGACTCACTATAGGGCGCGGCCGCAGAATTCGAGCTCGGTACCCGGGATCTCGAGGCCAGATCGTGCTTCGATATCGATCGTTTGGcaggcgctgggaccgcgggcggacaaggatgggtccgcgcggGgcCgcCTCTCTCccccgaggccccggacctcggcggctgctcctccccgtcgtcctcccgctgctgctgctgctgttgttggcgccgccgggctcgggcgccggggccagccggccgccccacctggtcttcttgctggcagacgacctaggctggaacgacgtcggcttccacggctcccgcatccgcacgccgcacctggacgcgctggcggccggcggggtgctcctggacaactactacacgcagccgctgtgcacgccgtcgcggagccagctgctcactggccgctaccagatccgtacaggtttacagcaccaaataatctggccctgtcagcccagctgtgttcctctggatgaaaaactcctgccccagctcctaaaagaagcaggttatactacccatatggtcggaaaatggcacctgggaatgtaccggaaagaatgccttccaacccgccgaggatttgatacctactttggatatctcctgggtagtgaagattattattcccatgaacgctgtacattaattgacgctctgaatgtcacacgatgtgctcttgattttcgagatggcgaagaagttgcaacaggatataaaaatatgtattcaacaaacatattcaccaaaagggctatagccctcataactaaccatccaccagagaagcctctgtttctctaccttgctctccagtctgtgcatgagccccttcaggtccctgaggaatacttgaagccatatgactttatccaagacaagaacaggcatcactatgcaggaatggtgtcccttatggatgaagcagtaggaaatgtcactgcagctttaaaaagcagtgggctctggaacaacacggtgttcatcttttctacagataacggagggcagactttggcagggggtaataactggccccttcgaggaagaaaatggagcctgtgggaaggaggcgtccgaggggtgggctttgtggcaagccccttgctgaagcagaagggcgtgaagaaccgggagctcatccacatctctgactggctgccaacactcgtgaagctggccaggggacacaccaatggcacaaagcctctggatggcttcgacgtgtggaaaaccatcagtgaaggaagcccatcccccagaattgagctgctgcataatattgacccAaacttcgtggactcttcaccgtgtcccaggaacagcatggctccagcaaaggatgactcttctcttccagaatattcagcctttaacacatctgtccatgctgcaattagacatggaaattggaaactcctcacgggctacccaggctgtggttactggttccctccaccAtctcaatacaatgtttctgagataccctcatcagacccaccaaccaagaccctctggctctttgatattgatcgggaccctgaagaaagacatgacctgtccagagaatatcctcacatcgtcacaaagctcctgtcccgcctacagttctaccataaacactcagtccccgtgtacttccctgcacaggacccccgctgtgatcccaaggccactggggtgtggggcccttggatgGGAAGCGGAGAGGGCAGAGGAAGTCTGCTAACATGCGGTGACGTCGAGGAGAATCCTGGACCTATTGAACAAGATGGATTGCACGCAGGTTCTCCGGCCGCTTGGGTGGAGAGGCTATTCGGCTATGACTGGGCACAACAGACAATCGGCTGCTCTGATGCCGCCGTGTTCCGGCTGTCAGCGCAGGGGCGCCCGGTTCTTTTTGTCAAGACCGACCTGTCCGGTGCCCTGAATGAACTGCAGGACGAGGCAGCGCGGCTATCGTGGCTGGCCACGACGGGCGTTCCTTGCGCAGCTGTGCTCGACGTTGTCACTGAAGCGGGAAGGGACTGGCTGCTATTGGGCGAAGTGCCGGGGCAGGATCTCCTGTCATCTCACCTTGCTCCTGCCGAGAAAGTATCCATCATGGCTGATGCAATGCGGCGGCTGCATACGCTTGATCCGGCTACCTGCCCATTCGACCACCAAGCGAAACATCGCATCGAGCGAGCACGTACTCGGATGGAAGCCGGTCTTGTCGATCAGGATGATCTGGACGAAGAGCATCAGGGGCTCGCGCCAGCCGAACTGTTCGCCAGGCTCAAGGCGCGCATGCCCGACGGCGAGGATCTCGTCGTGACCCATGGCGATGCCTGCTTGCCGAATATCATGGTGGAAAATGGCCGCTTTTCTGGATTCATCGACTGTGGCCGGCTGGGTGTGGCGGACCGCTATCAGGACATAGCGTTGGCTACCCGTGATATTGCTGAAGAGCTTGGCGGCGAATGGGCTGACCGCTTCCTCGTGCTTTACGGTATCGCCGCTCCCGATTCGCAGCGCATCGCCTTCTATCGCCTTCTTGACGAGTTCTTCTGAtgtaggatttcagggaggctagaaaacctttcaattggaaCCAAACGATCGATATCGAAGCACATTGTGGATCCGCTCTAGAGTCGACCTGCAGGCATGCAAGCTTGCGGCCGCGTATTCTATAGTGTCACCTAAATAGCATGGCGTAATCATGGTCATAGCTGTTTCCTGTGTGAAATTGTTATCCGCTCACAATTCCACACAACATACGAGCCGGAAGCATAAAGTGTAAAGCCTGGGGTGCCTAATGAGTGAGCTAACTCACATTAATTGCGTTGCGCTCACTGCCCGCTTTCCAGTCGGGAAACCTGTCGTGCCAGCTGCATTAATGAATCGGCCAACGCGCGGGGAGAGGCGGTTTGCGTATTGGGCGCTCTTCCGCTTCCTCGCTCACTGACTCGCTGCGCTCGGTCGTTCGGCTGCGGCGAGCGGTATCAGCTCACTCAAAGGCGGTAATACGGTTATCCACAGAATCAGGGGATAACGCAGGAAAGAACATGTGAGCAAAAGGCCAGCAAAAGGCCAGGAACCGTAAAAAGGCCGCGTTGCTGGCGTTTTTCCATAGGCTCCGCCCCCCTGACGAGCATCACAAAAATCGACGCTCAAGTCAGAGGTGGCGAAACCCGACAGGACTATAAAGATACCAGGCGTTTCCCCCTGGAAGCTCCCTCGTGCGCTCTCCTGTTCCGACCCTGCCGCTTACCGGATACCTGTCCGCCTTTCTCCCTTCGGGAAGCGTGGCGCTTTCTCATAGCTCACGCTGTAGGTATCTCAGTTCGGTGTAGGTCGTTCGCTCCAAGCTGGGCTGTGTGCACGAACCCCCCGTTCAGCCCGACCGCTGCGCCTTATCCGGTAACTATCGTCTTGAGTCCAACCCGGTAAGACACGACTTATCGCCACTGGCAGCAGCCACTGGTAACAGGATTAGCAGAGCGAGGTATGTAGGCGGTGCTACAGAGTTCTTGAAGTGGTGGCCTAACTACGGCTACACTAGAAGAACAGTATTTGGTATCTGCGCTCTGCTGAAGCCAGTTACCTTCGGAAAAAGAGTTGGTAGCTCTTGATCCGGCAAACAAACCACCGCTGGTAGCGGTGGTTTTTTTGTTTGCAAGCAGCAGATTACGCGCAGAAAAAAAGGATCTCAAGAAGATCCTTTGATCTTTTCTACGGGGTCTGACGCTCAGTGGAACGAAAACTCACGTTAAGGGATTTTGGTCATGAGATTATCAAAAAGGATCTTCACCTAGATCCTTTTAAATTAAAAATGAAGTTTTAAATCAATCTAAAGTATATATGTTTAAACTTGGTCTGACAGTTACCAATGCTTAATCAGTGAGGCACCTATCTCAGCGATCTGTCTATTTCGTTCATCCATAGTTGCCTGACTCCCCGTCGTGTAGATAACTACGATACGGGAGGGCTTACCATCTGGCCCCAGTGCTGCAATGATACCGCGAGACCCACGCTCACCGGCTCCAGATTTATCAGCAATAAACCAGCCAGCCGGAAGGGCCGAGCGCAGAAGTGGTCCTGCAACTTTATCCGCCTCCATCCAGTCTATTAATTGTTGCCGGGAAGCTAGAGTAAGTAGTTCGCCAGTTAATAGTTTGCGCAACGTTGTTGCCATTGCTACAGGCATCGTGGTGTCACGCTCGTCGTTTGGTATGGCTTCATTCAGCTCCGGTTCCCAACGATCAAGGCGAGTTACATGATCCCCCATGTTGTGCAAAAAAGCGGTTAGCTCCTTCGGTCCTCCGATCGTTGTCAGAAGTAAGTTGGCCGCAGTGTTATCACTCATGGTTATGGCAGCACTGCATAATTCTCTTACTGTCATGCCATCCGTAAGATGCTTTTCTGTGACTGGTGAGTACTCAACCAAGTCATTCTGAGAATAGTGTATGCGGCGACCGAGTTGCTCTTGCCCGGCGTCAATACGGGATAATACCGCGCCACATAGCAGAACTTTAAAAGTGCTCATCATTGGAAAACGTTCTTCGGGGCGAAAACTCTCAAGGATCTTACCGCTGTTGAGATCCAGTTCGATGTAACCCACTCGTGCACCCAACTGATCTTCAGCATCTTTTACTTTCACCAGCGTTTCTGGGTGAGCAAAAACAGGAAGGCAAAATGCCGCAAAAAAGGGAATAAGGGCGACACGGAAATGTTGAATACTCATACTCTTCCTTTTTCAATATTATTATAAGCATTTATCAGGGTTATTGTCTCATGAGCGGATACATATTTGAATGTATTTAGAAAAATAAACAAATAGGGGTTCCGCGCACATTTCCCCGAAAAGTGCCACCTGACGTCTAAGAAACCATTATTATCATGACATTAACCTATAAAAATAGGCGTATCACGAGGCCCTTTCGTC**
